# Long‐term priming of hypothalamic microglia is associated with energy balance disturbances under diet‐induced obesity

**DOI:** 10.1002/glia.24217

**Published:** 2022-05-23

**Authors:** María del Mar Fernández‐Arjona, Ana León‐Rodríguez, Jesús M. Grondona, María D. López‐Ávalos

**Affiliations:** ^1^ Instituto de Investigación Biomédica de Málaga‐IBIMA Málaga Spain; ^2^ Grupo de investigación en Neuropsicofarmacología, Laboratorio de Medicina Regenerativa Hospital Regional Universitario de Málaga Málaga Spain; ^3^ Departamento de Biología Celular, Genética y Fisiología, Facultad de Ciencias Universidad de Málaga, Campus de Teatinos Málaga Spain

**Keywords:** high fat diet, hypothalamus, MHCII, microglia priming, neuraminidase, neuroinflammation, obesity

## Abstract

Exposure of microglia to an inflammatory environment may lead to their priming and exacerbated response to future inflammatory stimuli. Here we aimed to explore hypothalamic microglia priming and its consequences on energy balance regulation. A model of intracerebroventricular administration of neuraminidase (NA, which is present in various pathogens such as influenza virus) was used to induce acute neuroinflammation. Evidences of primed microglia were observed 3 months after NA injection, namely (1) a heightened response of microglia located in the hypothalamic arcuate nucleus after an in vivo inflammatory challenge (high fat diet [HFD] feeding for 10 days), and (2) an enhanced response of microglia isolated from NA‐treated mice and challenged in vitro to LPS. On the other hand, the consequences of a previous NA‐induced neuroinflammation were further evaluated in an alternative inflammatory and hypercaloric scenario, such as the obesity generated by continued HDF feeding. Compared with sham‐injected mice, NA‐treated mice showed increased food intake and, surprisingly, reduced body weight. Besides, NA‐treated mice had enhanced microgliosis (evidenced by increased number and reactive morphology of microglia) and a reduced population of POMC neurons in the basal hypothalamus. Thus, a single acute neuroinflammatory event may elicit a sustained state of priming in microglial cells, and in particular those located in the hypothalamus, with consequences in hypothalamic cytoarchitecture and its regulatory function upon nutritional challenges.

## Main Points

- Ventricular inflammation drives long‐lasting priming of hypothalamic microglia.
- Response of primed hypothalamic microglia to challenges such as HDF and obesity is increased.
- Past inflammatory events alter hypothalamus and energy balance regulation.

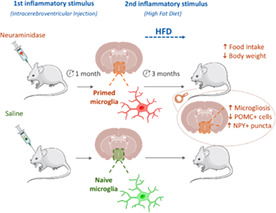

## 1 INTRODUCTION

Microglial cells are considered the main representative of the immune system within the brain parenchyma (Kettenmann et al., 2011; Prinz et al., 2014). They are endowed with an array of receptors which serve as sensors of the brain milieu, allowing the surveillant role of these cells. Microglia activate upon the detection of a wide variety of stimuli, and adopt a phenotype that can range along a gradient from pro‐inflammatory to anti‐inflammatory. After activation, microglia engage different functions aimed to the removal of pathogens, damaged cells, cellular debris, or pathogenic protein aggregates, and finally the restoration of homeostasis (Gomez‐Nicola & Perry, 2014; Li & Barres, 2018). Besides, microglia are important players in other physiological processes such as neurogenesis or synaptic pruning, thus contributing to the reorganization of synaptic networks (Allen & Lyons, 2018; Tremblay et al., 2011). If activation occurs, once the activating stimulus disappears and homeostasis is re‐established, microglia returns to the surveillant or resting state. However, in recent years a novel pseudo‐resting state of microglia has been described: after a first activating stimulus, microglia may acquire a primed state, that makes them more sensitive to subsequent stimuli, upon which they will execute an exacerbated response (Neher & Cunningham, 2019; Norden et al., 2015; Purisai et al., 2007). Primed microglia can give rise to a severe neuroinflammation, which may even lead to neurodegeneration and neurological disorders (Lisboa et al., 2016; Perry & Holmes, 2014; Tay et al., 2017; Yirmiya et al., 2015). Therefore, knowing the insights of microglial priming is of great relevance.

The factors able to induce microglial priming are diverse. It has been reported that age is one of such factors, but other inflammatory signals may also be so. In fact, any severe neuroinflammatory factor (e.g., microbes invading the nervous system, traumatisms and strokes) could be regarded as a potential priming stimulus. We have previously described a neuroinflammation model in which the intracerebroventricular (ICV) administration of the enzyme neuraminidase (NA) in rodents causes an acute neuroinflammatory process. We established that a single dose of 500 mU of NA administered ICV in rats or mice provoked conventional symptoms of neuroinflammation such as peripheral cells infiltration, activation of resident microglia and astrocytes, increased levels of mediators such as cytokines, complement system activation (Fernandez‐Arjona et al., 2017; Fernandez‐Arjona et al., 2019a, 2019b; Granados‐Durán et al., 2015; Granados‐Durán et al., 2016), and blood brain barrier permeability (unpublished data). Other symptoms included myelin vacuolation (Granados‐Durán et al., 2017) and ependymal cell damage (Fernandez‐Arjona et al., 2021; Granados‐Durán et al., 2016). After 2–3 weeks, most signs of inflammation have disappeared, and animals seem completely recovered (Granados‐Durán et al., 2015, and unpublished results), thus supporting the acute nature of the inflammatory process. Larger doses of NA used in the past provoked more severe symptoms: hydrocephalus with a massive loss of ependymal lining (Grondona et al., 1996). Thus, adjusting the dose of NA we can model hydrocephalus (if using a high dose) or neuroinflammation (if using lower doses). Our previous works (references above) using a low dose of 500 mU of NA demonstrate the validity of this model to reproduce an acute neuroinflammatory process. The rationale of using NA as inflammatory stimulus lies in the fact that this enzyme is part of a variety of microbes able to invade the central nervous system (CNS), for example, mumps virus (Love et al., 1985), several strains of bacteria producing meningitis (O'Toole et al., 1971), and even a highly widespread virus such as the influenza virus, which sometimes provokes neurologic complications (Antoon et al., 2021; Glaser et al., 2012; Maricich et al., 2004; Shinya et al., 2011; Steininger et al., 2003; Yildizdas et al., 2011). NA has been related to the infection and/or dispersion mechanism of these pathogens (Rossman & Lamb, 2011). Various aspects of NA‐induced inflammation have been described during the previous years (Fernandez‐Arjona et al., 2019a; Fernandez‐Arjona et al., 2021; Granados‐Durán et al., 2015; Granados‐Durán et al., 2016; Granados‐Durán et al., 2017). For instance, NA is able to activate microglial cells acting primarily through the pattern receptor toll‐like receptor 4 (TLR4) (Fernandez‐Arjona et al., 2019a). However, the question remains if, after NA‐induced inflammation, microglia might have acquired a primed state. Moreover, not only the nature of the priming stimulus is relevant, but also the time span the primed state may last, what could jeopardize neural functions in later stages of life. Therefore, here we aimed to find out if microglia acquire a primed state after NA‐induced inflammation, and if such state is maintained for a long period time.

On the other hand, it has been shown that a high fat diet (HFD) is also an inflammatory stimulus, so that traces of mild inflammation occur in different brain structures, including the hypothalamus, even after only few days of HFD consumption (De Souza et al., 2005; Milanski et al., 2009; Spencer et al., 2019). The mediobasal portion of the hypothalamus is of particular relevance for the regulation of energy balance, and lies in a privileged location where the blood brain barrier is more permeable, thus allowing the access of peripheral nutritional cues. In fact, hypothalamic microglia have been proposed as hypothalamic sensors able to orchestrate an inflammatory response in this area (Valdearcos et al., 2017). Hypothalamic inflammation has been shown to interfere with feeding and energy balance regulation, altering hypothalamic circuitry and leading to body weight dysregulation, including obesity (De Luca et al., 2020; Horvath et al., 2010; Reis et al., 2015; Thaler et al., 2012; Valdearcos et al., 2015; Yi et al., 2017; Zhang et al., 2008).

Given the prevalence of diets rich in fats in the population of developed and developing countries, here we design a paradigm picturing a neuroinflammatory process provoked by NA followed by a short‐term HFD few months later. In this situation, the primed state of hypothalamic microglia and their response to the HFD were investigated. A second experimental approach envisioned a scenario of obesity (which is known to concur with a mild central and peripheral inflammation) months after an acute neuroinflammatory process provoked by NA. Diet induced obesity imposes a challenge to the hypothalamus aimed to the regulation of feeding, body weight and energy balance, which might be altered after a past event of inflammation. The potential consequences of hypothalamic microglial priming on the regulation of feeding and body weight were investigated in obese mice.

## 2 MATERIALS AND METHODS

### 2.1 Animals

Three‐months old male wistar rats were used for *Experiment 1*. Male mice (C57BL/6J) of the same age were used for *Experiments 2* and *3*. Animals were housed under a 12 h light/dark cycle, at 23°C and 60% humidity, with food and water available ad libitum. Animal care and handling was performed according to the guidelines established by Spanish legislation (RD 53/2013) and the European Union regulation (Directive 2010/63/EU). All procedures performed were approved by the ethics committee of Universidad de Málaga and Consejería de Agricultura, Ganadería, Pesca y Desarrollo Sostenible, Junta de Andalucía (Ref. 04/10/2018/145). All efforts were made to minimize the number of animals used and their suffering.

### 2.2 Experiment 1

**FIGURE 1:**
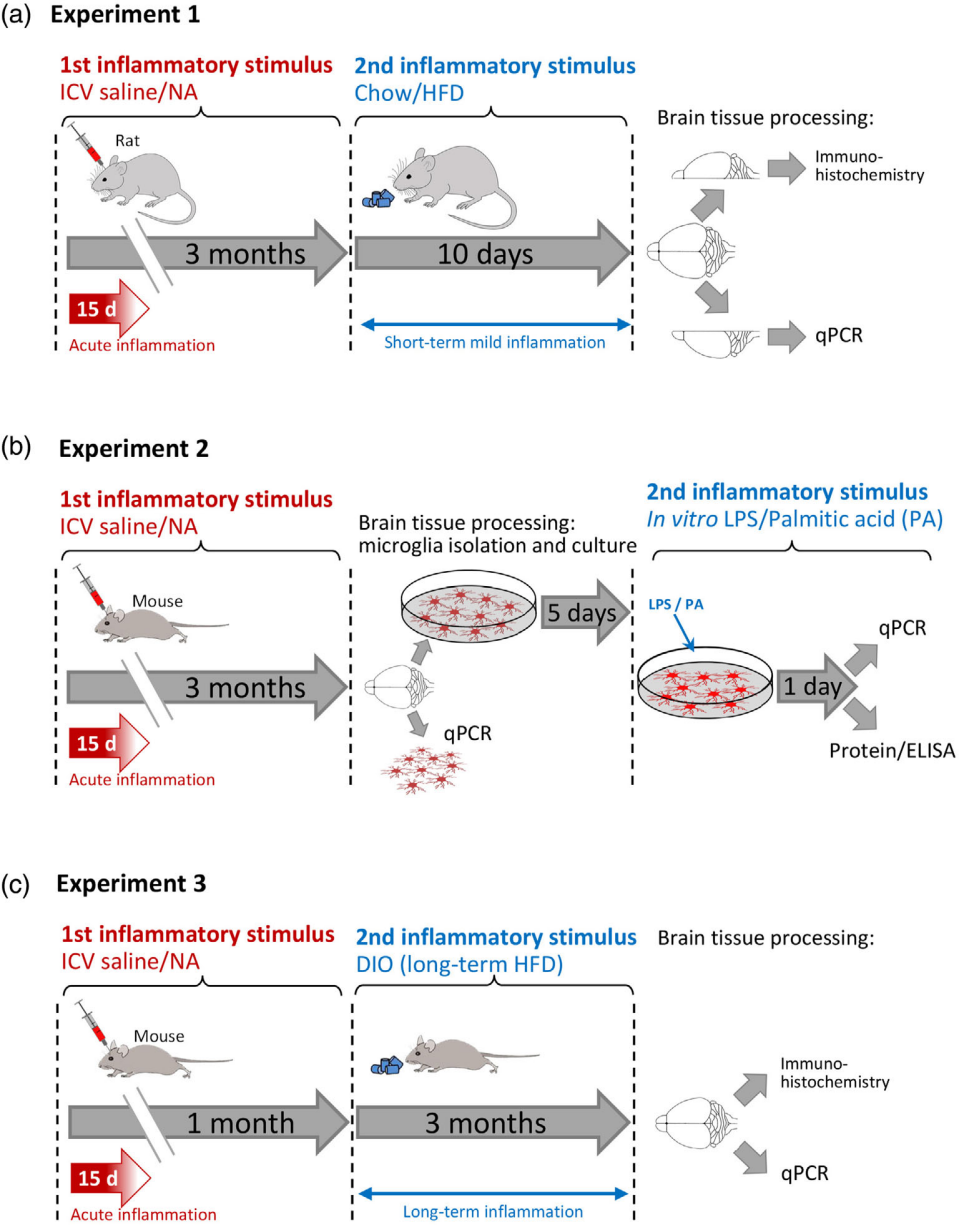
Scheme of the experiments designed to explore microglia priming at long term. (a) Experiment 1 was carried out with rats, which were applied a first neuroinflammatory stimulus consisting in the ICV injection of NA, followed by a second stimulus applied 3 months later, consisting in feeding with HFD for 10 days, which is known to induce mild hypothalamic inflammation. (b) Experiment 2 was carried out in mice, which received the same first stimulus as before. Gene expression was studied in isolated microglia 3 months later. In addition, in this case a second stimulus (consisting in LPS or palmitic acid) was applied in vitro to the isolated microglial cells. In both experiments, the response of microglial cells to the second stimulus was analyzed. (c) Experiment 3 was carried out in mice, with the aim of studying the possible influence of NA‐induced acute inflammation on the long‐term regulation of weight and food intake in a situation of obesity provoked by a HFD. The inflammatory state of the hypothalamus was studied by immunohistochemistry and qPCR

Male rats were ICV injected (see below) with neuraminidase (NA; first inflammatory stimulus; Figure 1a) or vehicle (saline solution). The acute inflammation thus provoked lasts about 2–3 weeks (Granados‐Durán et al., 2015). The rats were maintained during three more months under standard housing conditions, caged in pairs. Then, half of the animals were switched to a HFD for 10 days (second inflammatory stimulus; Figure 1a), while the other half remained with a standard chow. During this time, the body weight of each animal and the diet consumed (per cage, that is, two rats with the same treatment) were recorded daily. Finally, the anesthetized rats underwent a cardiac perfusion with heparinized saline. The brains were extracted and cut along the sagittal plane. The right half was immersed in 4% paraformaldehyde (PFA) and processed for immunohistochemistry. The left side was further dissected out, and the hypothalamus was immediately frozen in liquid nitrogen for later RNA isolation and qPCR. Six rats were included in each experimental group: Sal‐Chow, NA‐Chow, Sal‐HFD, and NA‐HFD.

### 2.3 Experiment 2

This experiment was designed with the aim of assessing isolated microglia. Because the method for isolation was established for mouse microglia (see Section 2.9 in Materials and Methods), Experiment 2 was carried out with mice. Male mice were ICV injected (see below) with NA (first inflammatory stimulus; Figure 1b) or vehicle (saline solution; sham‐ICV). Some mice were left without any injection (no‐ICV). A total of 5–7 mice were included in each experimental group: no‐ICV, sham‐ICV, NA‐ICV. Similarly as in Experiment 1, the mice were allowed to recover from inflammation, and maintained up to 3 months after the ICV with no further treatment. Then, each mouse was anesthetized, its brain extracted, and microglia isolated. Microglial cells were (i) immediately used for RNA isolation or (ii) placed in culture for 5 days, and then exposed to a second inflammatory stimulus. Palmitic acid (PA) was chosen as this second stimulus for being a long‐chain saturated fatty acid known to be a microglia activator present in HFDs (Valdearcos et al., 2014; Yanguas‐Casas et al., 2018). Lipopolysaccharide (LPS) was used as well, as positive control of the pro‐inflammatory response of microglia. Some cultures were simultaneously treated with both stimuli. After 24 h under stimulation, the media were collected, and microglia harvested for either qPCR or protein extraction.

### 2.4 Experiment 3

Male mice were ICV injected (see below) with NA (first inflammatory stimulus; Figure 1c) or vehicle (saline solution). The mice were allowed to recover from the acute inflammation during 1 month, and caged in threesomes. Then, all the animals were switched to a HFD for 3 months, so that to provoke a diet induced obesity (DIO; second inflammatory stimulus). During this time, the body weight of each animal and the diet consumed (per cage, that is, three mice with the same treatment) were recorded weekly. A total of 12 mice were included in each experimental group: Sal‐DIO and NA‐DIO. Finally, the anesthetized mice were processed by either method: (i) cardiac perfusion with 4% PFA for brain tissue immunohistochemistry; (ii) cardiac perfusion with heparinized saline, brain extraction, and hypothalamus dissection for RNA isolation and qPCR.

### 2.5 ICV injection

An acute neuroinflammatory process was provoked in rats/mice by a single injection of the enzyme NA within the right lateral ventricle of the brain (Gomez‐Roldan et al., 2008; Grondona et al., 1996). Sham animals were injected with 0.9% sterile saline. ICV injection procedure was performed as previously described (Fernandez‐Arjona et al., 2019a; Granados‐Durán et al., 2015). Briefly, the rodents were anesthetized with ketamine/xylazine solution (80 and 12 mg/kg, respectively) and positioned in a stereotaxic frame. In the case of rats, a scalp incision along the sagittal midline was performed to access the skull, and the bone was perforated with a drill in the following coordinates: 0.5 mm posterior and 1.4 mm lateral from Bregma (Paxinos & Watson, 2007). NA from *Clostridium perfringens* (Sigma‐Aldrich, N3001) dissolved in 0.9% sterile saline was administered by a single injection, 3.5 mm below the dura mater, with the aid of a pump; a dose of 500 mU (in 20 μl) of NA was injected during 10 min, at a rate of 2 μl/min. This ICV injection procedure was fine‐tuned for mice. In this case, stereotaxic coordinates were 0.1 mm posterior and 0.9 mm lateral from Bregma (Franklin & Paxinos, 2008). The amount of NA injected was 75 mU in 1 μl, administered 2.0 mm below the dura mater, and perfused during 5 min at a rate of 0.2 μl/min.

### 2.6 Immunohistochemistry

The brains of 4% PFA perfused animals were extracted and post‐fixed during 24 h. They were sectioned in a vibratome, obtaining series of free‐floating 40 μm coronal sections. Serial sections including the hypothalamus were labeled with different primary antibodies: rabbit anti‐IBA1 (1:500; Wako 19‐19741), rabbit anti‐GFAP (1:1000; Sigma G‐9269), rabbit anti‐POMC (1:1000; Phoenix Europe GMBH H‐029_30), rabbit anti‐NPY (1:1000; Sigma N‐9528), goat anti‐IL‐1β (1:500; R&D Systems AF‐501‐NA), rat anti‐Ly6c (1:1000; Bio‐Rad MCA2389GA), and mouse anti‐MHC Class II (MHC II, 1:500; Novusbio NB100‐64959).

To quench endogenous peroxidase, sections for immunoperoxidase procedures were incubated in 10% methanol and 3% hydrogen peroxide in PBS during 45 min. After washings, nonspecific binding sites were saturated with PBT solution (0.3% bovine serum albumin, 0.3% Triton X‐100 in PBS pH 7.3). The sections were then placed in primary antibody solution for overnight incubation at 4°C. The following morning the sections were washed and incubated with biotinylated secondary antibody (1:1000; biotin‐conjugated goat anti‐rabbit, Pierce), at room temperature for 1.5 h. The avidin‐biotin‐complex amplification system (1:250; ABC Thermo Fisher Scientific) was used afterwards, incubated also at room temperature for 45 min. Finally, the peroxidase activity was developed with a solution of 0.05% diaminobenzidine and 0.03% hydrogen peroxide in PBS, for 10 min. After thorough washes, the sections were then mounted onto gelatin‐coated slides and air‐dried. Sometimes toluidine blue was used to counterstain the brain tissue. Then, slices were dehydrated in graded ethanol, cleared in xylene, and coverslipped with Eukitt mounting medium. All antibodies were diluted in PBT and washes were performed with PBS pH 7.3.

For immunofluorescent staining, after saturating with PBT solution, the primary antibodies were mixed and incubated simultaneously (overnight at 4°C). Alexa fluor secondary antibodies were used (Alexa fluor 647 anti‐rabbit IgG for anti‐IBA1, Alexa fluor 594 anti‐mouse IgG for anti‐MHC II, Alexa fluor 488 anti‐goat IgG for anti‐IL‐1β, and Alexa fluor 488 anti‐rat IgG for anti‐Ly6c; all from Invitrogen). Depending on the primary antibodies used, secondary antibodies were selected accordingly, mixed and diluted in PBT to a final concentration of 1:1000, and incubated for 1.5 h at room temperature in the dark. After washes, coverslides were mounted with buffered glycerol containing 10% of the anti‐fading agent Mowiol 4–88 (Calbiochem/EMD Chemicals). The immunofluorescently labeled sections were analyzed with a confocal microscope (Leica, SP5 II, Wetzlar, Germany).

### 2.7 Quantification of immunostained hypothalamus sections

To quantify the percentage of IBA1‐positive cells also stained for MHC II in hypothalamic arcuate nucleus, one series of sections per animal was double‐stained with rabbit anti‐IBA1 and mouse anti‐MHC II by immunofluorescence. Three to four sections per animal were used, and two to three images per section of the arcuate nucleus were acquired with a confocal microscope and a HC PL APO CS2 ×40/1.30 oil objective (Leica SP5 II) and used for cell counts. Counting was carried out by the same researcher and blind to the experimental group.

Positive cell counts were performed in GFAP, IBA1 and POMC immunoperoxidase stained sections including the hypothalamus. In the case of NPY, immunoreactive puncta were quantified. A series of brain sections from each animal was used for each counting, including at least six sections, 240 μm apart. In rats, sections spanned approximately from Bregma −1 to −3 mm, and in mice from Bregma 0 to −2 mm. Each section was photographed using ×10 or ×20 objective lenses. The arcuate hypothalamic nucleus was enclosed and cut using the ImageJ software (https://imagej.nih.gov); positive cells were manually counted, and referred to the enclosed area. For NPY‐labeled puncta, a particle size cut was established using ImageJ software, so only those puncta >1 μm^2^ were included; counting was done with the tools of ImageJ software. All counts were done blind to the experimental group.

### 2.8 Morphological analysis of microglial cells

For the morphological analysis of individual microglial cells, high‐resolution image acquisition was carried out on IBA1‐labeled samples. Digital color images were obtained with an Olympus VS120 scanner microscope. High‐resolution images (pixel side = 115 nm) of the hypothalamus including the arcuate nucleus were captured using the UPLSAPO ×60 oil immersion objective. A multiplane virtual‐Z mode allowed to capture 20 images (1 μm thick) in a 20 μm tissue section depth, which were later combined to obtain a single high‐quality image, including detailed magnification of ramified processes of the cells. Each acquired image was a TIFF file of 96 ppi. Two sections per animal were scanned for this analysis.

Microglial cells morphology was analyzed using *FIJI* free software (http://fiji.sc/Fiji). A comprehensive explanation of the procedure is available elsewhere (Fernández‐Arjona et al., 2017). Briefly, the TIFF image was subjected to RGB channel separation, and the blue channel was chosen for the analysis (as the brown color from oxidized diaminobenzidine is enhanced in this channel). The image was then filtered to soften the background, the contrast was enhanced, and then it was transformed to obtain an image in eight‐bit gray scale. The appropriate threshold (previously established by trials and then applied to all images) was later adjusted and the image was then transformed in a binary image. From this binary image, microglial cells were randomly selected as follows: (i) random selection starting from the area next to the third ventricle toward the brain parenchyma, (ii) no overlapping with neighboring cells, (iii) complete nucleus and branches (at least apparently). A total of 10 cells in the arcuate nucleus per animal were selected, a process done blinded to treatment. Each cell image was then processed to obtain a *filled image* and an *outline image*. Manual edition was required: some pixels were cleared to separate ramifications pertaining to neighboring cells, while some pixels were added to join processes belonging to the selected cell. This skilled step was carefully done under the view of the original image of the cell, with caution to avoid any bias.

The *filled image* and the *outline image* thus obtained were used to measure morphometric parameters of microglial cells. Fifteen different parameters were measured using *FracLac* for *ImageJ* sofware (Karperien et al., 2013). Those parameters were the following: *fractal dimension* (*D*), *lacunarity* (Λ), *cell area, convex hull area*, *density, cell perimeter, convex hull perimeter, roughness, convex hull of span ratio, cell circularity, convex hull circularity, diameter of the bounding circle, maximum span across the convex hull*, the *ratio maximum/minimum convex hull radii*, and the *mean radius*. The detailed description of each parameter is available in Table S2 and Figure S1.

### 2.9 Microglia isolation and in vitro stimulation

Microglial cells were isolated from mice which had been previously injected with NA or saline. For isolation, magnetic‐activated cell sorting (MACS) technology and the Adult Brain Dissociation Kit, both from Miltenyi Biotec (reference 130‐107‐677) were used, following to the manufacturer's protocol. Whole brain was chopped into small pieces and incubated with a pre‐warmed Enzyme Mixture (included in the kit) in agitation at 37°C. The tissue was further dissociated mechanically (passing through 19G and 21G needles). The sample was then filtered through a cell strainer (100 μm). Cellular debris and red blood cells were removed with the aid of the Debris Removal Solution and the Red Blood Cell Removal Solution respectively. MACS MicroBead separation were used to isolate CD11b‐positive microglial cells by using anti‐CD11b antibody coated magnetic beads (MicroBeads #130‐093‐634). The cells/beads suspension was loaded onto a MACS Column (Miltenyi Biotec), which was placed in the magnetic field of a MACS Separator (Miltenyi Biotec). After removing the column from the magnetic field, the magnetically retained CD11b‐positive cells were eluted as the selected cell fraction. The purity of these microglial isolates, checked by immunocytochemistry, was usually ≈ 98%. The average yield was ≈ 500,000 cells/mouse brain.

A fraction of the isolated cells (≈ 100,000 cells) was immediately used for RNA isolation. The rest of microglial cells were seeded in 12 multi‐well plates (≈ 35,000 cells/well) in 1 ml of DMEM‐F12 medium (Gibco; 11320033), supplemented with 10% fetal bovine serum (FBS, Sigma‐Aldrich; F7524) and 1% penicillin/streptomycin (Sigma‐Aldrich; P4333). After seeding, cells were left in culture during 5 days to allow their stabilization after isolation and their adhesion to the culture plate. Then, microglial cultures were stimulated by the addition of palmitic acid (PA; Sigma, P5585; 100 μM), lipopolysaccharide (LPS; InvivoGen, 13I06‐MM; 1 μg/ml), which was used here as a positive control of the pro‐inflammatory response of microglia, or both together. Twenty‐four hours after the addition of PA and/or LPS, the culture media were harvested, and the cells were used either for RNA isolation or for protein extraction.

### 2.10 Palmitic acid solution for in vitro stimulation

A palmitic acid:bovine serum albumin (PA:BSA) solution was prepared as follows. Palmitic acid (Sigma, P5585) was dissolved in ethanol at a concentration of 100 mM, in a warm bath at 42°C. Concurrently, DMEM‐F12 medium containing 1% FBS and 2% fatty‐acid free bovine serum albumin (BSA; Sigma, A4612) was prepared in sterile conditions. The 100 mM PA ethanol solution was then added to the warmed medium (42°C) to obtain a 10x PA:BSA stock solution (containing 1 mM PA). To avoid PA precipitation, this addition was done very slowly, mixing and maintaining the temperature at 42°C. Once mixed, this stock was kept at 42°C during 20 min to favor the coupling between PA and BSA. The same procedure was done in parallel, starting with ethanol without any PA, to prepare a 10x no‐PA:BSA stock, to be added to control non‐stimulated cultures. PA was employed for being a long‐chain saturated fatty acid known to be a microglia stimulant present in HFD (Valdearcos et al., 2014; Yanguas‐Casas et al., 2018). PA was added to a final concentration of 100 μM (from a 10x PA:BSA stock; see above). When both stimuli were used, LPS was added 1 h before PA, so that microglial cells were already under pro‐inflammatory stimulation when encountering PA. Control cultures were added a preparation of BSA without PA (10x no‐PA:BSA stock).

### 2.11 RNA isolation and qPCR

Total RNA was isolated using TRIzol reagent (Invitrogen, 15596‐026). One milliliter of reagent was added to about 100 mg of tissue (dissected hypothalamus). When RNA was isolated from microglia cultures, the culture medium was removed, the cells washed with cold PBS, and 0.5 ml of TRIzol reagent was added to the well (≈35,000 cells). RNA was isolated following manufacturer's instructions. Finally, the RNA was resuspended in a volume of ≤50 μl of RNase‐free water. The concentration of RNA was measured in a NanoDrop microvolume spectrophotometer (NanoDrop 1000, Thermo Fisher Scientific). The A260/280 ratio of the isolated RNA was usually about 1.8.

RNA samples were diluted with RNase‐free water in order to bring them to a similar range of RNA concentration. cDNA synthesis from isolated total RNA was performed using the SuperScriptTM III First‐Strand Synthesis (Invitrogen). The reaction mix was prepared according to the manufacturer's protocol, and RNA was added to have ≈500 ng in a final volume of 20 μl. In these conditions, the resulting cDNA concentration should be equivalent to 25 ng RNA/μl. cDNA was stored at –20°C.

For real‐time quantitative PCR (qPCR), the hot‐start reaction mix FastStart Essential DNA Green Master (Roche), based on SYBR Green I fluorescence dye, was used. qPCR reactions were prepared following manufacturer's instructions. Forward and reverse primers were used at a final concentration of 0.4 μM, and cDNA at 40 ng/20 μl (amount established after preliminary trials). The qPCR reaction was carried out in a LightCycler 96 Instrument (Roche). The information obtained (amplification curves, melting curves and crossing points, CP, or cycle threshold, Ct) for each transcript was processed using the software provided with the LightCycler equipment. The expression of each target gene was relativized to the level of expression of the housekeeping gene glyceraldehyde 3‐phosphate dehydrogenase (GAPDH). Primers to target the mRNA of genes of interest were designed using the program Primer3 (https://primer3.org/). Target genes sequences were obtained from the GenBank at NCBI Reference Sequence (https://www.ncbi.nlm.nih.gov/). Data of the primers used is available in Table S1.

### 2.12 Protein extraction and ELISA for IL‐1β

After in vitro stimulation of isolated mouse microglia (Experiment 2), culture media were harvested from each well, and the plate placed on ice. The cells were washed with cold PBS, and 200 μl of cold lysis buffer (RIPA buffer with a cocktail of protease inhibitors, Roche) were added to each well. After 10 min, the solution was aspirated up and down repeatedly for cell detachment and lysis, and then collected. Protein concentration was determined by the bicinchoninic acid assay, and protein extracts stored at −20°C until ELISA.

To measure the amount of IL‐1β released by microglial cells, ELISA was carried out in the previously collected culture media. ELISA was done following the manufacturer's indications (DuoSet ELISA, R&D Systems), with some modifications aimed to increase detectability. Briefly, rat anti‐mouse IL‐1β (capture antibody) was used at 10 μg/ml, biotinylated goat anti‐mouse IL‐1β (detection antibody) was used at 0.5 μg/ml, and streptavidin‐HRP was used at 1:40. In these conditions, IL‐1β levels in culture media were under the detection limit (9 pg/ml). Therefore, only data from cultured microglia lysates were obtained.

### 2.13 Statistical analysis

The data shown in graphs are presented as the mean ± *SEM*. Statistical analysis of data was done with SPSS Statistics or with Microsoft Excell softwares. The Kolmogorov–Smirnov normality tests, along with the Levene homoscedasticity test, were used to verify if data could be analyzed by parametric methods. One‐way analysis of variance (ANOVA) was used to compare the mean values of the parameters, followed by the Bonferroni test for pairwise comparisons. In the case of non‐parametric data, the Kruskal‐Wallis test was performed, and pairwise comparisons were done with Tamhane pairwise multiple comparisons. In experiments with only two experimental groups, comparisons were carried out with the Student's *t* test. In all cases, differences were considered significant when a *p*‐value <.05 was obtained.

## 3 RESULTS

### 3.1 Compensatory food intake and weight gain are apparently normal in rats that suffered NA‐induced inflammation

**FIGURE 2:**
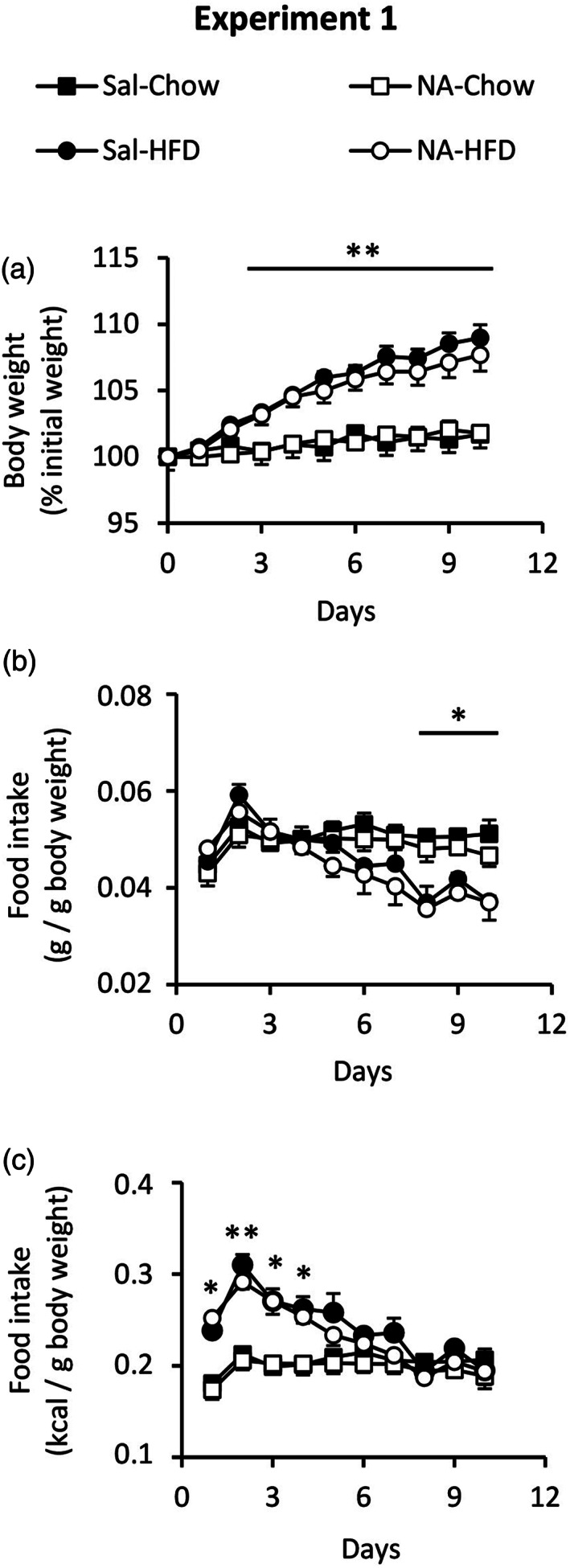
Body weight and food intake of rats during the HFD treatment (Experiment 1). Rats injected ICV with NA or saline (Sal) 3 months before, were fed a HFD or standard chow during 10 days. Body weight and food intake were recorded daily. (a) The weight of the animals fed a HFD increased significantly from day 2 compared with those fed a standard chow (*p* < .01). (b, c) Regarding food intake, rats fed a HFD ate progressively less amount of the diet (b), reaching such decrease a statistical difference by day 8. When food intake is expressed as caloric intake (c), it is evidenced that the progressive reduction in HFD consumption results in the normalization of the caloric intake, which from day 5 is equal to that of rats fed a standard diet. No difference in food intake was observed between the animals previously injected with saline and NA. Data presented are the mean ± *SEM*. Body weight is of *n* = 6 rats per group. Food intake corresponds to that of two rats kept in the same cage, and three cages; therefore, *n* = 3. *p < .05; **p < .01

With the aim of investigating the maintenance of the primed state by microglial cells in the long term, a first inflammatory stimulus was applied to rats, consisting in the ICV injection of NA; control rats were injected with saline. NA provokes a transient inflammation, which has been previously described (Granados‐Durán et al., 2015). Three months later, a second inflammatory stimulus was applied, consisting in this case in a HFD during a short period of time (10 days), so that to induce hypothalamic inflammation without reaching obesity; control rats continued under a standard chow diet (Figure 1a). Weight and food intake were monitored during these 10 days (Figure 2). Although during the first days all animals ate the same amount of diet, those who took HFD progressively reduced their intake (Figure 2b), reflecting a compensatory mechanism to account for the higher caloric content of the HFD. As a result, after 6 days the caloric intake of the rats taking either diet was the same (Figure 2c). No differences in food intake were observed in the rats treated previously with NA compared with those injected with saline. As expected, the animals under HFD gained considerably more weight than those that continued with the standard chow (Figure 2a). Although no statistical difference in weight gain occurred between animals treated with NA compared with those injected with saline, a slight separation of the weight gain curves could be observed by the end of the HFD treatment, with the control animals pretreated with saline (Sal‐HFD) gaining slightly more weight than those pretreated with NA (NA‐HFD).

### 3.2 NA‐provoked inflammation induces a long‐term increase in MHC II‐positive microglia in the basal hypothalamus

**FIGURE 3:**
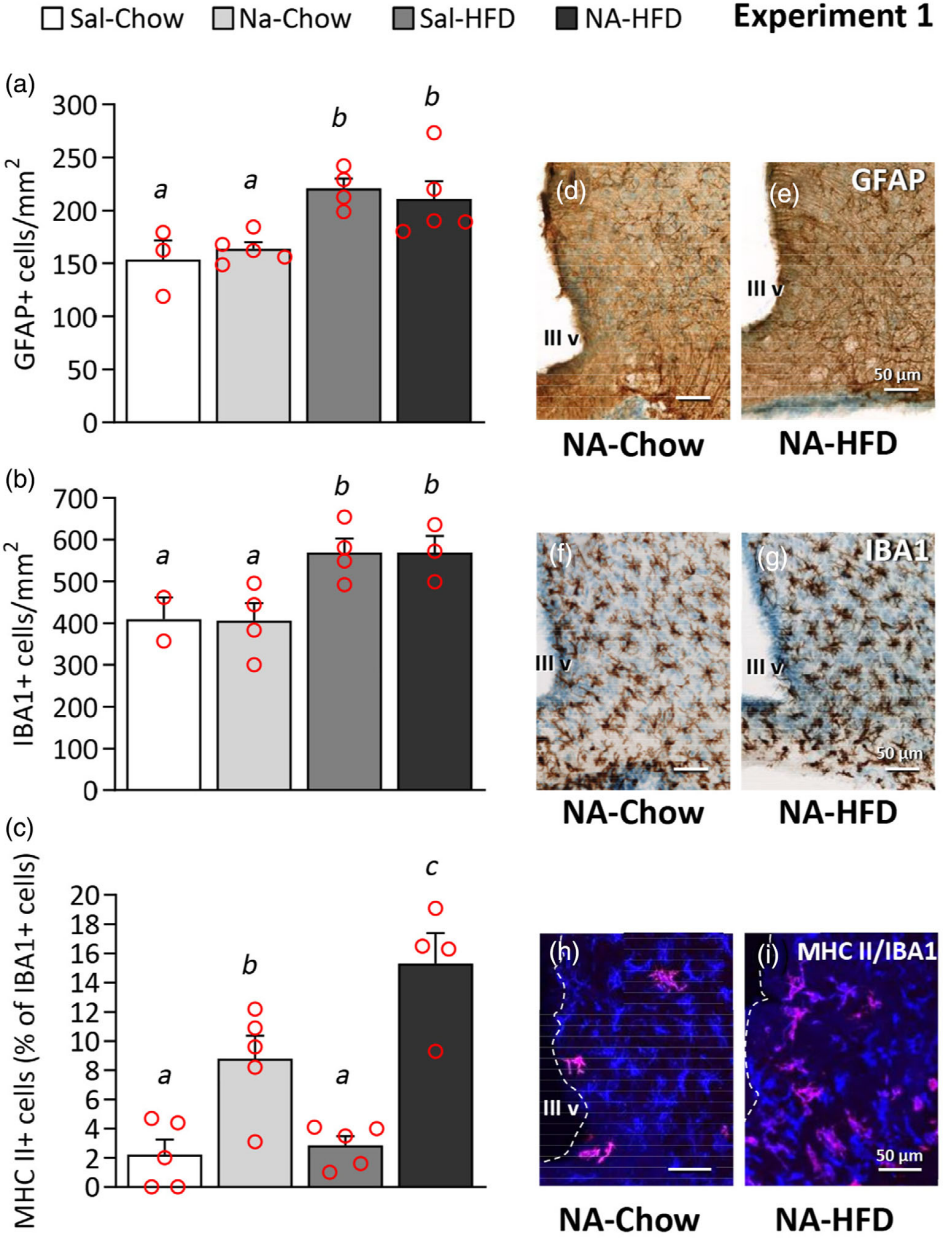
GFAP, IBA1, and MHC II cell counts in the arcuate nucleus of rats treated with two inflammatory stimuli applied 3 months apart. First, the rats were ICV injected with saline (Sal) or NA, and 3 months later they were fed a standard chow or a HFD (Experiment 1). (d–i) Brain sections containing the arcuate nucleus were immunostained for GFAP (d, e), IBA1 (f, g), or double stained for IBA1 (blue) and MHC II (red) (h, i). (a–c) The graphs present the number of cells labeled with GFAP (a) and IBA1 (b) in the arcuate nucleus, as well as the percentage of IBA1‐positive cells that also express MHC II (c). The histograms show the mean ± *SEM* of *n* = 3–6 animals. The letters *a–c* above the bars indicate if a statistical difference exists between groups: The same letter means no difference; different letter means statistical difference (*p* < .05). III v: third ventricle

The HFD has been reported to be an inflammatory stimulus in the hypothalamus (Thaler et al., 2012). Therefore, the hypothalamus was studied for signs of inflammation. As previously described, 10 days of HFD provoked a glial response, with increased numbers of both astrocytes (Figure 3a) and microglia (Figure 3b) in the arcuate nucleus (ARC). However, no differences were observed between animals pretreated with NA or saline.

The major histocompatibility complex class II (MHC II), which is expressed by antigen presenting cells, has been reported as a marker of microglial activation and/or priming (Holmin & Mathiesen, 1999; Ling et al., 2004; Loane et al., 2014; Muccigrosso et al., 2016; Trzeciak et al., 2019). Therefore, we investigated the presence of MHC II‐positive cells in the basal hypothalamus. Double staining with IBA1 confirmed that all MHC II‐positive cells in the hypothalamus were microglia, or at least monocyte/macrophage derived cells (as IBA1 labels all these cell types). The percentage of MHC II‐positive cells within the IBA1‐positive population was calculated (Figure 3c); it was very low in control animals pretreated with saline and, interestingly, did not increase upon the second stimulus with HFD. However, the percentage of MHC II‐positive cells in the ARC of rats pretreated with NA was significantly higher even 3 months after NA injection, a percentage that further increased when the animals were switched to the HFD (Figure 3c,h,i).

**FIGURE 4:**
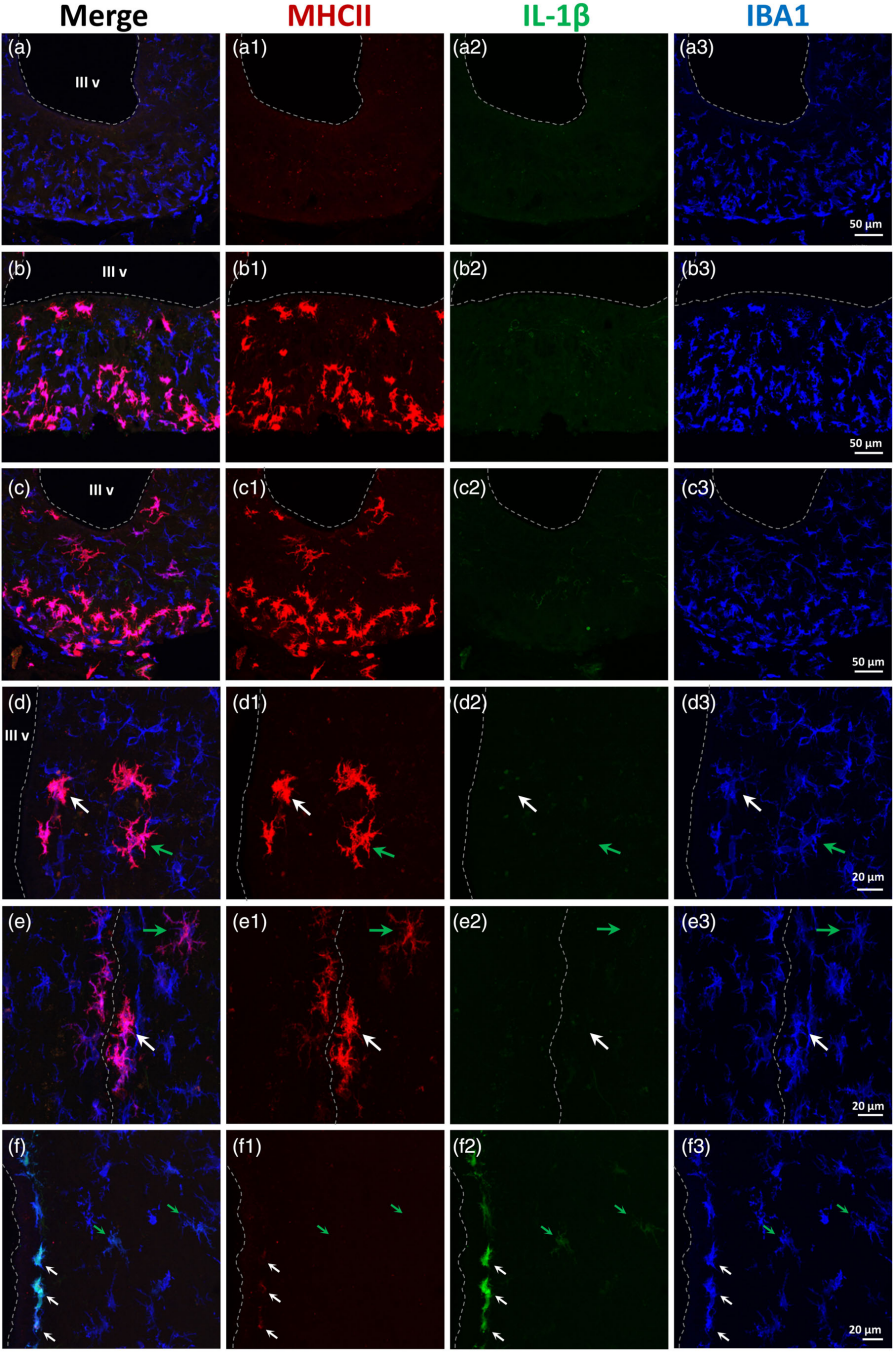
The ICV injection of NA induces the presence of MHC II‐positive microglia in the hypothalamus in the long term. Triple immunofluorescence staining for MHC II (red), IL‐1β (green), and IBA1 (blue). (a) Median eminence of an animal injected with saline, where no MHC II‐positive cells are found. (b, c) median eminence of rats injected with NA, and 3 months later fed a HFD (b) or the standard chow (c). IBA1/MHC II‐positive cells are abundant in the median eminence, as well as in the nearby arcuate nucleus. None of these cells labels for IL‐1β. (d). The arcuate nucleus of a NA‐injected HFD‐fed rat, where similar IBA1/MHC II‐positive (and IL‐1β‐negative) cells appear. Some of these cells show an activated morphology (white arrow), while others are more ramified (green arrow). (e) In locations dorsal to the arcuate nucleus (mid‐dorsoventral level of the third ventricle; the opposing ventricular walls appear in contact, and are delimited by a dashed white line) it is possible to find IBA1/MHC II‐positive cells in some animals. These cells locate close to the ventricular surface, frequently under the ependymal epithelium. They may present an activated morphology (white arrow) or a ramified one (green arrow). (f) The wall of the third ventricle (dorsal level) of an animal injected with NA and sacrificed 12 h later (sample from other study used here as positive control for IL‐1β staining). The induction in the expression of IL‐1β by microglial cells (particularly those located close to the ventricular surface) is well documented in this case of neuroinflammation. The degree of IL‐1β expression, as well as the compaction, are higher in subependymal microglia (white arrows) and milder in microglia located deeper in the parenchyma (green arrows). None of these cells expresses MHC II, except for a faint label in those subependymal cells that are apparently more activated (white arrows). In all images, the broken line outlines the ventricular surface. III v: Third ventricle

**FIGURE 5:**
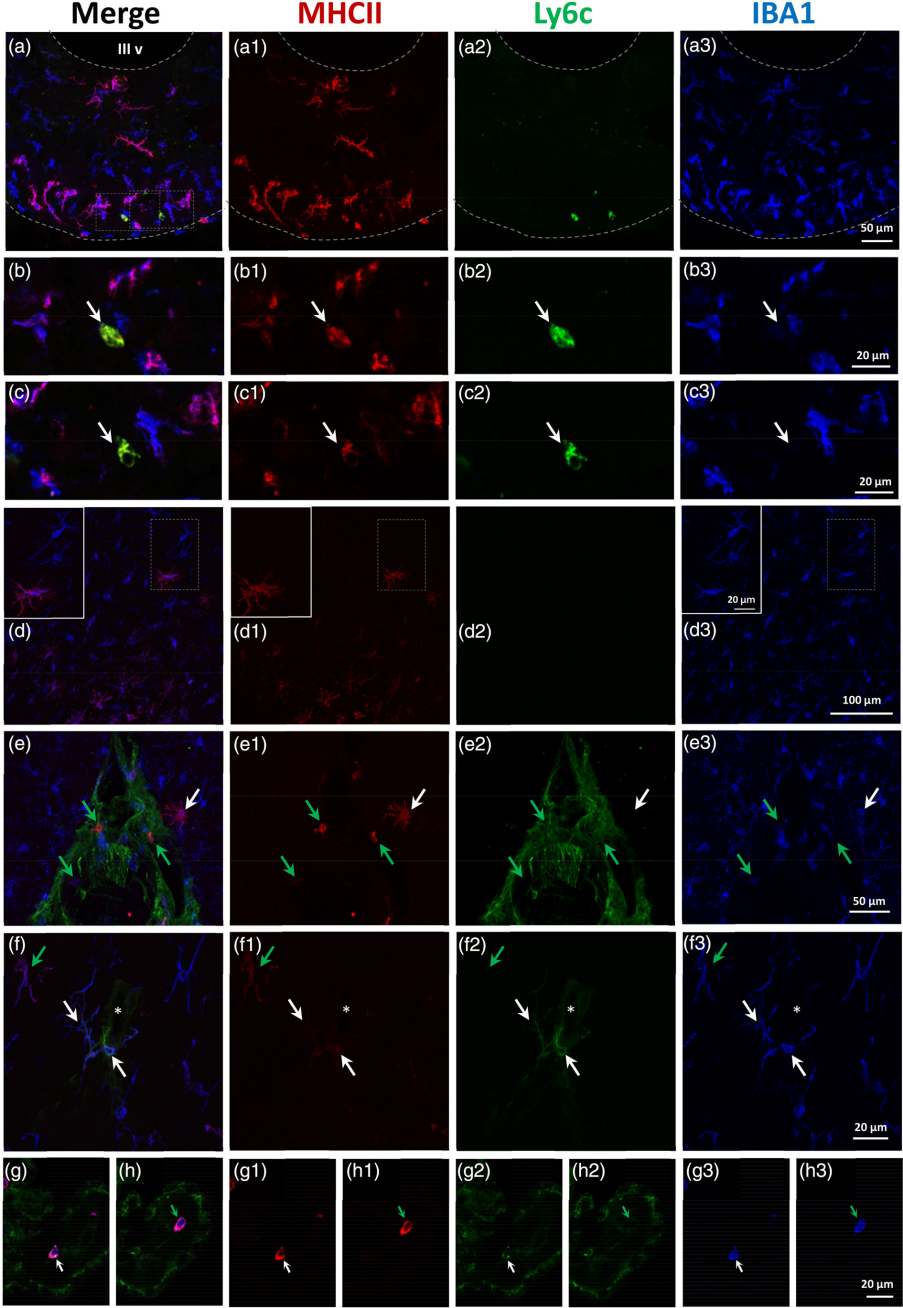
The IBA1/MHC II‐positive cells in NA‐injected rats do not label with the marker Ly6c. Triple immunofluorescence staining for MHC II (red), Ly6c (green) and IBA1 (blue). (a) Three months after the ICV‐injection, the median eminence of NA‐injected rats present abundant IBA1/MHC II‐positive cells; however, none of these cells labels with Ly6c antibody (a2). (b, c) Higher magnifications of rectangles in (a) showing single Ly6c‐positive cells, which lie in the external layer of the median eminence; a single confocal plane is shown. These cells are also MHC II‐positive (b1, c1), but IBA1‐negative (b3, c3). (d) In the cortex close to the injection site, it is possible to find IBA1‐positive microglia which are MHC II‐positive (d1), but always Ly6c‐negative (d2). Among these cells ramified morphology is most common. The inset is a higher magnification of the squared area, showing a ramified MHC‐positive microglia as well as MHC‐II negative cells. (e) Inferior dural venous sinus located between the cerebral hemispheres, close to the injection site. Dura mater and the wall of blood vessels are Ly6c‐positive. Round MHC II‐positive cells (most of which are also IBA1‐positive) are associated to dura mater meninges (green arrows). The white arrow points to a cortical microglial cell which is MHC II‐positive (e1) but Ly6c‐negative (e2), and presents a ramified morphology. (f) A magnified view of a region of the corpus callosum close to the injection site, showing two rare IBA1/MHC II‐positive cells (white arrows) which are also Ly6c‐positive (f3). These cells are in close association to a blood vessel (asterisk) which, as expected, labels with Ly6c. The green arrow points to a more common IBA1/MHC II‐positive cell with no Ly6c label. (g, h) The choroid plexus within the lateral ventricle offers an opportunity to find examples of peripheral cells labeled with the markers used here. Thus, the white arrow in (g) points a cell which is positive with the three markers, while the green arrow in h points a cell that is IBA1/MHC II‐positive but not labeled by Ly6c. In addition, the choroid plexus cells themselves are Ly6c‐positive. III v: Third ventricle

MHC II‐positive cells in the basal hypothalamus were located in the nervous parenchyma, close to the ventricular surface (Figure 4e) or at deeper locations (Figure 3h,i). These cells were particularly abundant in the median eminence, especially in its external layer (farther from the ventricle) and usually appeared as compact cells with short and thick ramifications. In this location, almost no MHC II‐positive cells were found in saline‐treated animals (Figure 4a), while they were frequent in NA‐treated rats, both fed standard chow (Figure 4b) or HFD (Figure 4c). MHC II‐positive cells were also found in the nearby ARC (Figure 3h,i; Figure 4d), some of them with a compact morphology (white arrow in Figure 4d) while others were more ramified (green arrow in Figure 4d). MHC II‐positive cells were sometimes found in locations dorsal to the ARC and close to the ventricular surface, even within the subependymal layer (white arrow in Figure 4e). Here, the degree of ramification of MHC II‐positive cells also ranged from high (green arrow in Figure 4e) to low (white arrow in Figure 4e). MHC II‐positive cells with a morphology proper of resting microglial cells, that is, with intricate, long and thin ramifications and small cell body, could be found in other locations, particularly in the cortex close to the injection site (Figure 5d; white arrow in Figure 5e). As in the basal hypothalamus, only a fraction of these IBA1‐positive microglial cells expressed MHC II (Figure 5d).

To explore the properties and identity of these MHC II/IBA1‐positive cells, triple immunostainings were performed with IL‐1β, a marker of the pro‐inflammatory activation of microglia (Figure 4). In the NA‐induced inflammation model, it is well characterized the induction of IL‐1β in microglial cells in the vicinity of the ventricular cavities, few hours after the injection of NA (Fernandez‐Arjona et al., 2017). Such activation was evident in samples from other experiments, where rats were sacrificed 12 h after the ICV‐injection of NA, used here as positive controls (Figure 4f). Subependymal microglia appears strongly stained for IL‐1β (white arrows in Figure 4f), and some microglia located in the periventricular nervous parenchyma is labeled as well (green arrows in Figure 4f). However, none of these cells stains for MHC II (arrows in Figure 4f1); if any label, only a slight staining is observed in those subependymal cells with the strongest IL‐1β label (white arrows in Figure 4f1). On the contrary, no IL‐1β was detected in any MHC II/IBA1‐positive cell from rats NA‐injected and sacrificed 3 months later, irrespective of receiving a normal chow or a HFD (Figure 4b2,c2,d2,e2). Thus, MHC II/IBA1‐positive cells do not appear to correspond to a canonical pro‐inflammatory activation.

On the other hand, and given the fact that MHC II/IBA1‐positive cells were particularly abundant in the median eminence, which is a highly vascularized zone, we explored the possibility of these cells being infiltrated monocytes/macrophages. Triple immunofluorescence staining was performed with the marker Ly6c (Figure 5), which is a membrane protein expressed by various immune cell types like monocytes/macrophages (Lee et al., 2013). As expected, Ly6c labeled endothelial cells of blood vessels (Figure 5e2; asterisk in Figure 5f3), and cells within the blood vessels of choroid plexus (Figure 5g2). The choroid plexus cells themselves were also Ly6c‐positive (Figure 5g2,h2). At the median eminence, however, none of the MHC II/IBA1‐positive cells stained for Ly6c (Figure 5a). Only scarce Ly6c‐positive cells were found in the external layer of the median eminence, where blood vessels are located (arrows in Figure 5b,c); besides, although these Ly6c‐positive cells were also MHC II‐positive (Figure 5b1.c1), they were morphologically different (small and not ramified) from the MHC II/IBA1‐positive population, and did not stain for IBA1 (arrows in Figure 5b3,c3). In addition, MHC II/IBA1‐positive cells found in other regions, like the cortex nearby the injection site, did not stain either for Ly6c (Figure 5d; white arrow in Figure 5e2). In all the samples analyzed, only two cells that were MHC II/IBA1‐positive and localized in the corpus callosum near the injection site, presented Ly6c staining (white arrows in Figure 5f2). They were associated to a Ly6c‐positive blood vessel (asterisk in Figure 5f). Thus, these cells might represent an example of the peripheral origin of some MHC II/IBA1‐positive cells; however, they seem to represent an uncommon event.

### 3.3 Morphological and fractal analysis uncovers a primed phenotype of hypothalamic microglia

**FIGURE 6:**
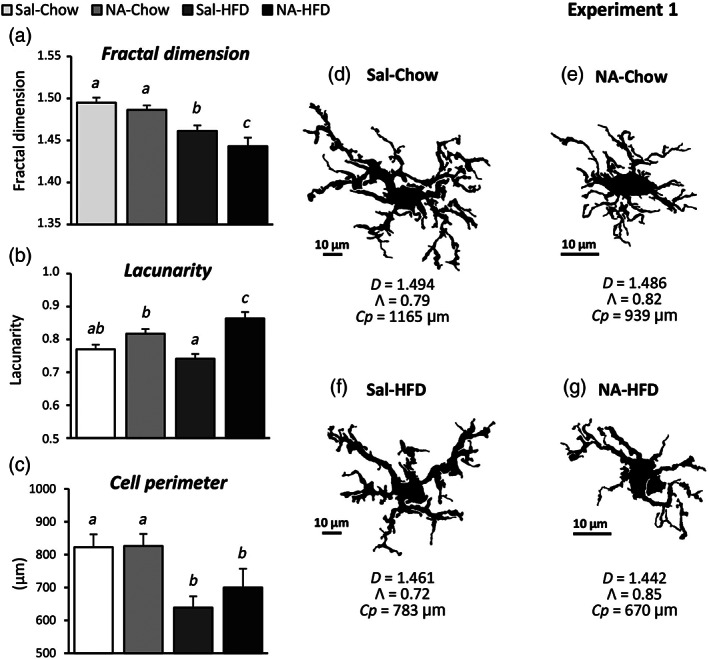
Morphological characterization of microglial cells in the arcuate nucleus of rats treated with two inflammatory stimuli applied 3 months apart. Rats were treated with two inflammatory stimuli (NA and HFD), applied 3 months apart, using saline (Sal) and standard chow, respectively as controls for those stimuli (Experiment 1). Brain sections were immunostained for IBA1. Images of IBA1‐positive microglia located in the arcuate nucleus were processed to obtain a binary image of the cell's profile; (d–g) some are shown here as examples. These processed images were used to measure morphometric parameters: (a) *fractal dimension*, (b) *lacunarity*, (c) *cell perimeter* (other parameters are shown in Data S1). The graphs represent the mean ± *SEM* of these parameters measured in *n* = 30–40 cells from each experimental group. Letters *a–c* above the bars denote if there is a statistical difference between groups: The same letter stands for no difference; different letters stand for a statistical difference (*p* < .05). (d–g) Images of representative arcuate nucleus microglial cells exposed to the different treatments. Their values of fractal dimension (*D*), lacunarity (*Λ*), and cell perimeter (*Cp*) are displayed below each cell

The morphological analysis of microglial cells has been proved to be a powerful tool to evaluate the activation state of these cells (Fernandez‐Arjona et al., 2017; Fernandez‐Arjona et al., 2019b). Individual microglial cells stained with IBA1 were sampled from the ARC. A total of 15 different morphological parameters (Figure S1 and Table S2) were measured in the image processed files of those cells; only three of them have been presented here for simplicity (Figure 6). Microglial *cell perimeter* decreased similarly upon HFD administration, irrespective of the prior ICV treatment, indicating the inflammatory impact of the HFD on hypothalamic microglia (Figure 6c). *Fractal dimension* (*D*; a higher *D* means a greater complexity of a pattern) behaved in a similar manner, decreasing only after HFD but not changing with the ICV‐treatment (Figure 6a). However, cells from NA‐HFD rats presented a lower *fractal dimension* than those from Sal‐HFD, suggesting that the previous treatment with NA enhances the microglial response to the HFD. Moreover, *lacunarity* (Λ; a higher Λ value implies heterogeneity, containing the cell profile many differently sized gaps or lacunas) was slightly higher (although not statistically significant) in microglia from NA‐injected animals compared with that of saline‐treated ones, and increased even more upon HFD treatment (Figure 6b), further supporting the results of *fractal dimension*. Therefore, although 3 months after the ICV injection of NA microglial morphology is apparently unaltered, however some parameters (*lacunarity* and *density*; Figure S2) reveal a subtle difference between NA‐ and saline‐treated animals. In addition, upon the HFD stimulus the morphological change of microglia from NA‐treated animals is further enhanced, compared with the change of microglia from saline‐treated animals, as revealed by some morphological parameters (*fractal dimension* and *lacunarity*; Figure 6a,b). In addition, the reported changes of microglia from NA‐treated animals are in accordance with more compact, more heterogeneous, de‐ramified cells, a morphology proper of more activated microglia.

The cellular profile of microglial cells chosen as examples from each experimental group allows to observe a more ramified morphology, thinner processes and a larger cell size in the Sal‐Chow cell (Figure 6d) compared with the NA‐Chow cell (Figure 6e). In addition, the Sal‐HFD cell (Figure 6f) shows less and thicker processes than the Sal‐Chow cell (Figure 6d), indicating the HFD activating effect on microglia. When comparing the Sal‐HFD cell (Figure 6f) with the NA‐HFD one (Figure 6g) it appears evident that this latter is smaller in size, has an enlarged cell body and is less ramified, all of them morphological signs of a higher degree of activation. Thus, upon HFD, rats that received an ICV‐injection of NA develop an enhanced microglial response in the hypothalamic ARC. This subjective evaluation of microglial morphology is however endorsed by the objective measurement of morphological parameters above presented.

### 3.4 Gene expression analysis reveals moderate signs of inflammation and microglia priming in the hypothalamus 3 months after NA‐induced inflammation

**FIGURE 7:**
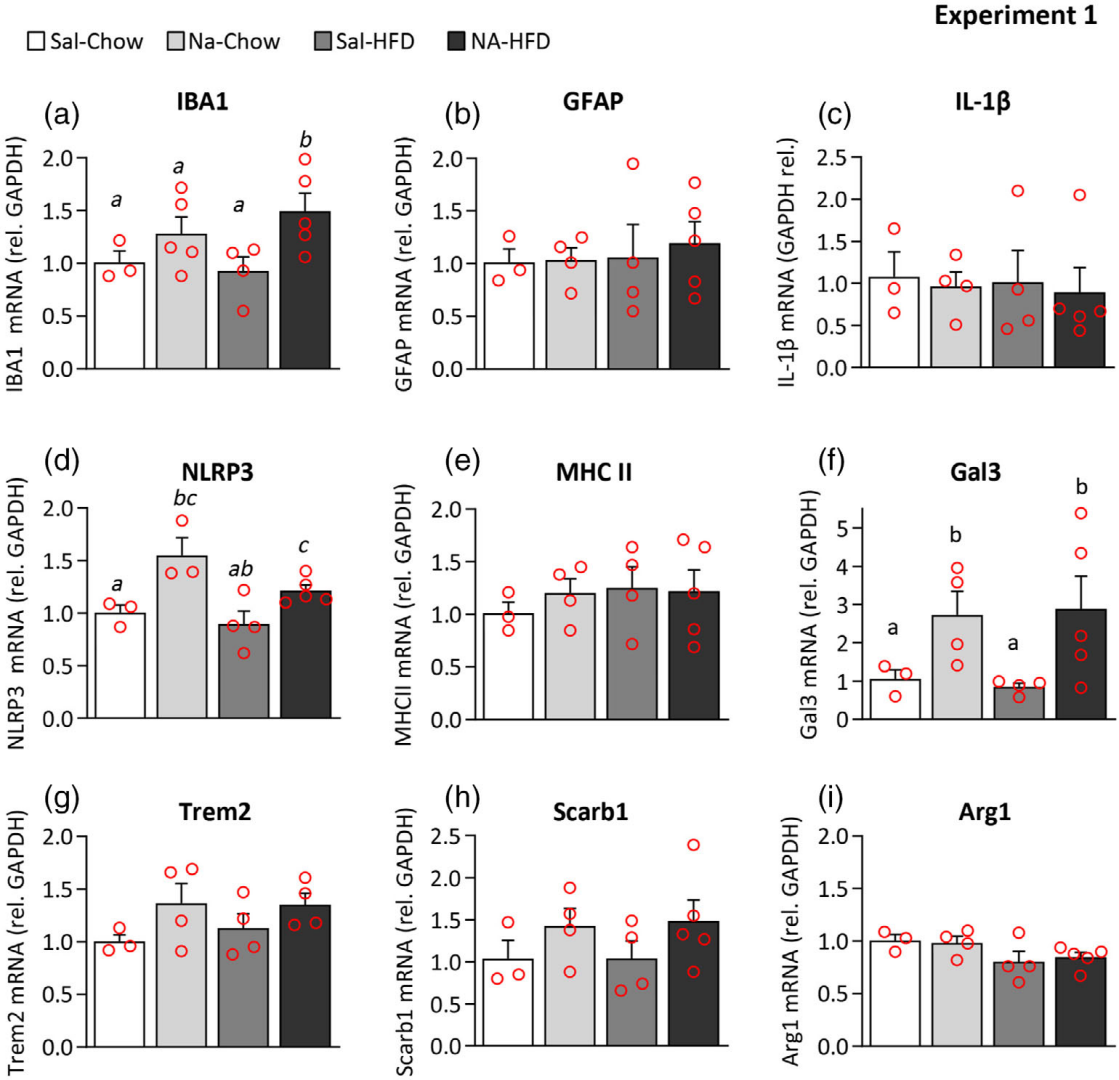
Quantification of gene expression in the hypothalamus of saline/NA and chow/HFD treated rats. The hypothalamus of rats that underwent exposure to two inflammatory stimuli separated in time (Experiment 1) was dissected out for RNA isolation and qPCR. The expression of genes related to neuroinflammation and microglial activation was quantified by qPCR, and their mRNA level expressed relative to GAPDH, and normalized to the control (Sal‐chow). (a–i) The histograms show the mean ± *SEM* of *n* = 3–5 animals per group, and the letters *a‐c* on top of the bars indicate the absence (if the same letter) or presence (if different letters) of a statistical difference between the compared groups (*p* < .05). The lack of any letter denotes that there is no statistical difference between any of the groups regarding that particular gene

The expression of a set of genes related to inflammation was examined in the hypothalamus of rats subjected to two inflammatory stimuli applied 3 months apart (Experiment 1). Three months after the injection of NA, and without the application of a second stimulus (NA‐chow), the expression of the astrocytic marker GFAP was similar to that of saline‐injected animals (Sal‐chow; Figure 7b), and the expression of IBA1 tended to be increased (NA‐chow vs. Sal‐chow) although was not statistically significant (Figure 7a). The pro‐inflammatory cytokine IL‐1β also remained unaltered (Figure 7c), as well as the antigen presenting molecule MHC II (Figure 6e). However, there was a significant increase in the expression of the inflammasome protein NLRP3 (Figure 7d) and of the inflammatory mediator galectin 3 (Gal3; Figure 7f). Other microglial receptors related to the function of clearing debris or noxious molecules, like triggering receptor expressed on myeloid cells 2 (Trem2) and scavenger receptor class B member 1 (Scarb1), showed a tendency, although not significant, to be overexpressed in the hypothalamus of NA‐treated rats (Figure 7g,h). The expression of arginase 1 (Arg1), an enzyme whose induction is associated to the alternative/anti‐inflammatory activation of microglia/macrophages, also remained unchanged (Figure 7i); a tendency (not‐significant) to its downregulation upon HFD‐treatment may be noted. Surprisingly, the exposure to a second inflammatory stimulus, namely a HFD for 10 days, did not alter the expression of most of these genes, that is, no differences were found between NA‐Chow and NA‐HFD groups. Only the expression of IBA1 in NA‐treated rats further increased when these were exposed to HFD (NA‐chow vs. NA‐HFD), but not so in saline‐injected rats (Sal‐chow vs. Sal‐HFD), suggesting a primed state of microglial cells in NA‐treated animals (Figure 7a). Notably, the expression of the cytokine IL‐1β, frequently used as a hallmark of a pro‐inflammatory activation, was similar in all the experimental groups (Figure 7c), which is in accordance with the immunostaining results (Figure 4). Of note, the expression of MHC II was also similar in all groups (Figure 7e) in spite of the results obtained by immunohistochemistry, that revealed a significant increase in the proportion of IBA1‐positive cells expressing MHC II in NA‐treated rats (Figure 3c; Figure 4a–c).

### 3.5 In isolated microglial cells, gene expression analysis and in vitro stimulation confirm a moderate state of priming 3 months after NA‐induced inflammation

**FIGURE 8:**
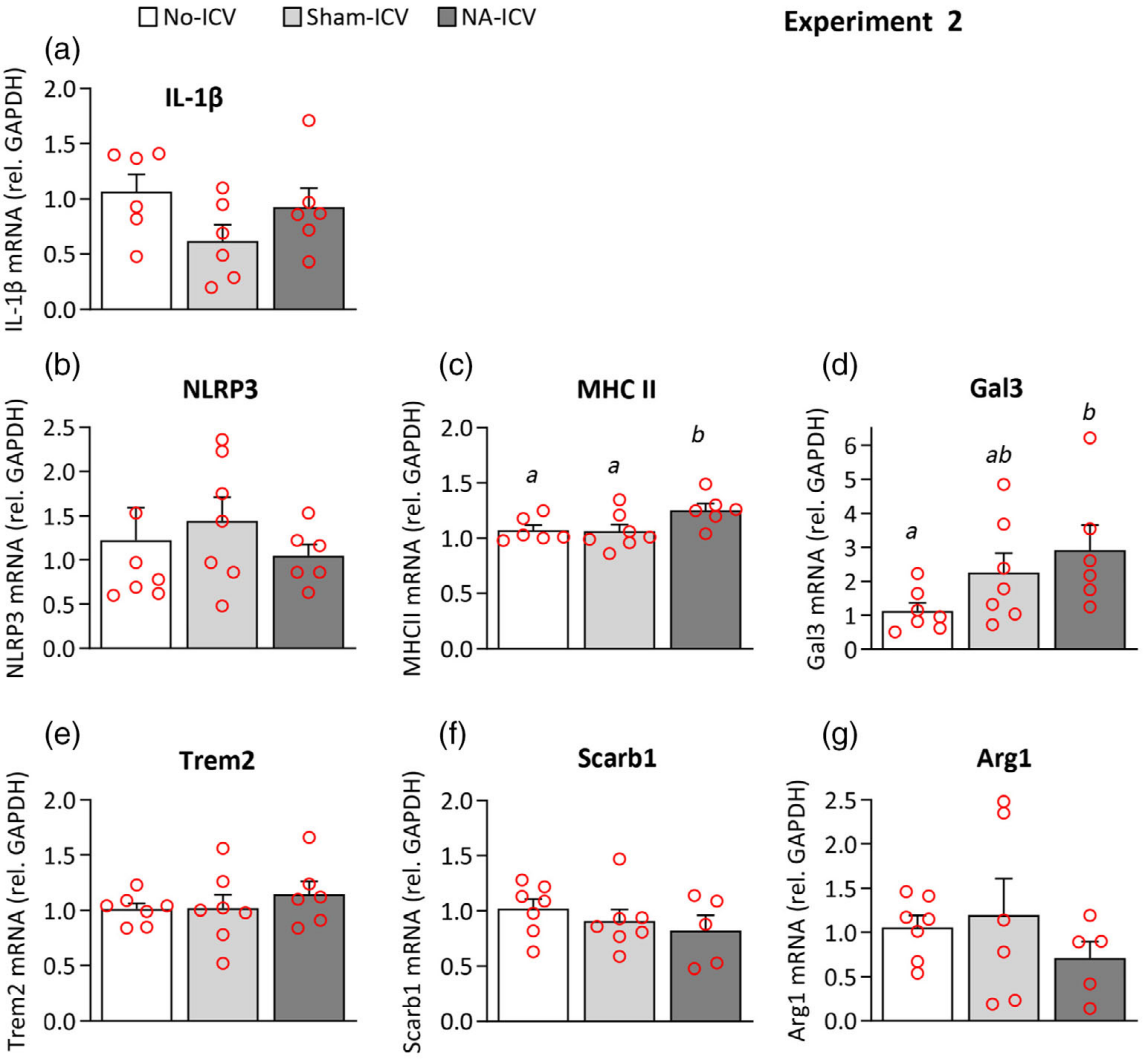
Quantification of gene expression in microglial cells isolated from mice exposed to a single neuroinflammatory stimulus 3 months before. Mice were ICV injected with NA (NA‐ICV), with saline (Sham‐ICV), and 3 months later their brains were extracted and microglial cells isolated from the whole brain (Experiment 2). Immediately after isolation, RNA was obtained, and the expression of genes related to microglial activation and priming was measured by qPCR. Microglia isolated from not injected mice (No‐ICV) were used as a reference. (a–g) The graphs show mRNA levels expressed relative to the housekeeping gene GAPDH. The bars in the histograms represent the mean ± *SEM* of *n* = 6–7 animals per group, and the letters *a–b* on top of the bars indicate the absence (if the same letter appears, or if no letter at all) or existence (if different letters appear) of a statistical difference (*p* < .05)

To more precisely evaluate the long‐term state of microglia after NA‐provoked inflammation, mice were ICV‐injected with NA (NA‐ICV) and microglia were isolated from the whole brain 3 months later (Experiment 2); control mice were ICV‐injected with sterile saline (Sham‐ICV). Gene expression was quantified in purified microglia isolated from NA or saline injected mice (Figure 8). To account for the possible impact of ICV surgery, a third experimental group consisting in non‐injected mice was processed along (No‐ICV). The microglial expression of IL‐1β, NLRP3, Trem2, Scarb1, and Arg1 was not significantly different between NA and saline injected microglia, nor there were differences compared with microglia from non‐injected mice (Figure 8a,b,e–g, respectively). However, MHC II was slightly overexpressed in microglia from NA‐treated mice (Figure 8c). Interestingly, the expression of Gal3 was induced in microglial cells, an induction possible owed not only to the ICV administration of NA but also to the ICV‐surgery itself (Figure 8d).

**FIGURE 9:**
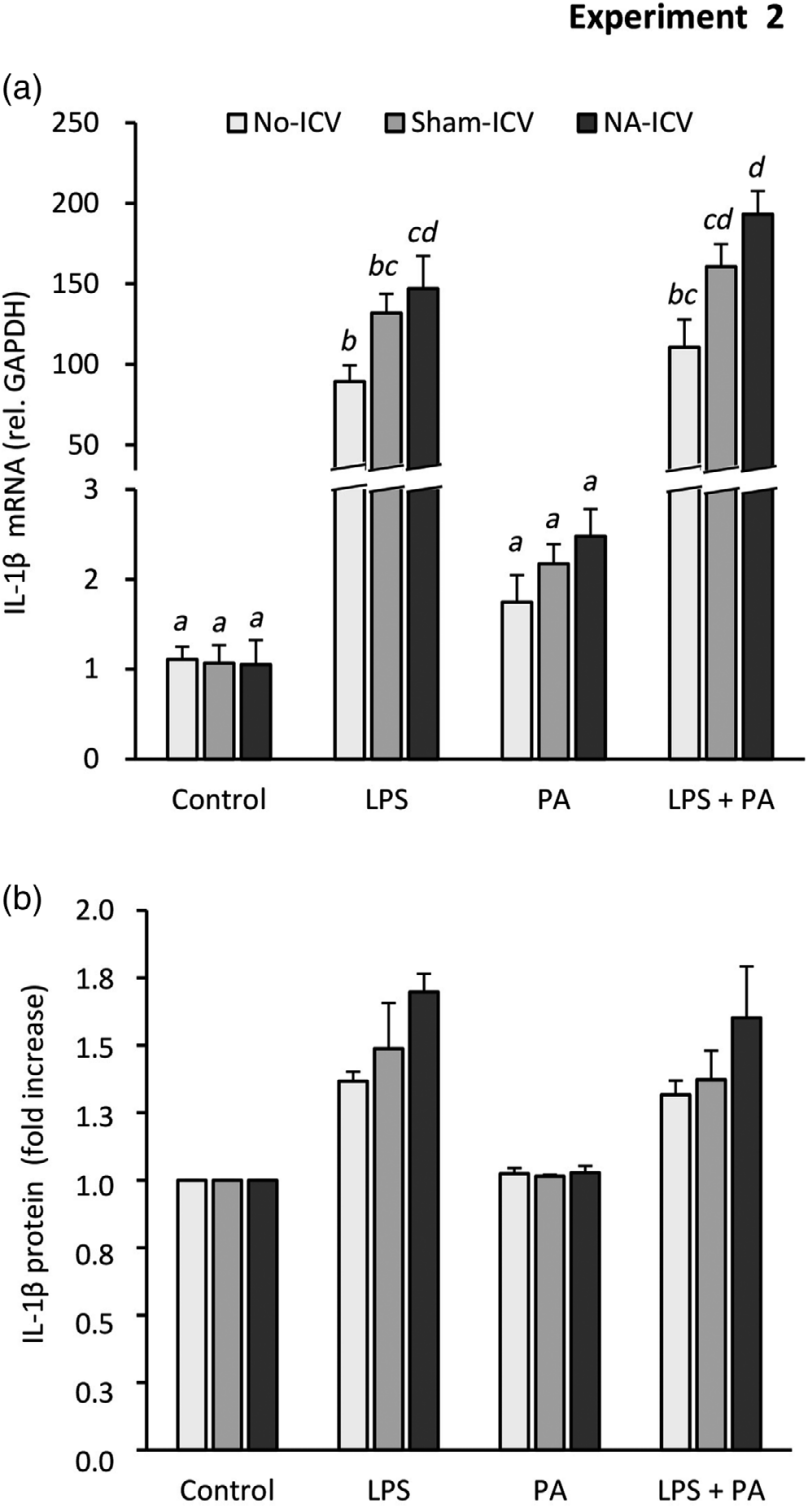
In vitro stimulation of microglial cells isolated from mice exposed to a neuroinflammatory stimulus 3 months before. Microglia were isolated from mice which received an ICV injection of NA (NA‐ICV), of saline (Sham‐ICV). After 5 days in culture, the cells were stimulated with 1 μg/ml lipopolisaccharide (LPS), 100 μM palmitic acid (PA) or both, during 24 h (see Experiment 2). The response of microglial cells was evaluated by the expression of IL‐1β, both by qPCR (a) and by ELISA (b) carried out on microglia lysates. Microglia isolated from not injected mice (No‐ICV) were used as a reference. The graphs show the mean ± *SEM* of *n* = 4–6 animals per group, and the letters on top of the bars (*a–d*) indicate the existence (if different letters) or the absence (if the same letter) of a statistically significant difference (*p* < .05). (b) The IL‐1β protein measured in microglia lysates is expressed as fold increase relative to control cultures. The mean amount of IL‐1β in lysates of control cultures was 0.45 ± 0.05 pg/μg of protein. The absence of letters on top of the bars indicates that there is no statistical difference between any of the culture conditions

A fraction of the isolated microglia was placed in culture and, after 5 days, they were in vitro stimulated to investigate if they developed an exacerbated response, a feature of primed microglia. Stimulation was performed with palmitic acid (PA, 100 μM), a long‐chain saturated fatty acid present in HFDs (Valdearcos et al., 2014; Yanguas‐Casas et al., 2018), and also with LPS (1 μg/ml), a well‐known and potent microglia stimulant which is an agonist of the receptor TLR4. Some cultures were stimulated with both LPS and PA simultaneously. After 24 h stimulation, the culture media and microglial cells were harvested to measure the induction of IL‐1β expression and secretion (as indicator of microglial activation). PA was unable to increase IL‐1β gene expression significantly (Figure 9a), although a tendency to increase (not statistically significant) could be observed when compared with control non‐stimulated cultures. On the contrary, LPS provoked a robust induction in the expression of IL‐1β. Interestingly, such induction was slightly higher in cells from NA‐ICV mice compared with Sham‐ICV mice, and also somewhat higher in microglia from Sham‐ICV animals compared with No‐ICV ones (Figure 9a). In the culture media, secreted IL‐1β was undetectable by ELISA, although it could be measured in cell lysates (Figure 9b). Although differences between groups were not statistically significant, IL‐1β protein levels are in accordance with gene expression results (Figure 9a), confirming the induction of IL‐1β by LPS but not so by PA, and also the greater extent of such induction in microglia isolated from NA‐treated mice. Thus, these results demonstrate that, under the same LPS stimulus, the response of microglial cells was slightly enhanced when they had been exposed to another inflammatory stimulus previously applied in vivo, namely NA or even the ICV‐surgery itself. Besides, the importance of the ICV procedure on the outcome of experiments aimed to evaluate the long‐term consequences of inflammation was also highlighted.

### 3.6 NA‐induced inflammation provokes a long‐term dysregulation of food intake and body weight under diet induced obesity conditions

**FIGURE 10:**
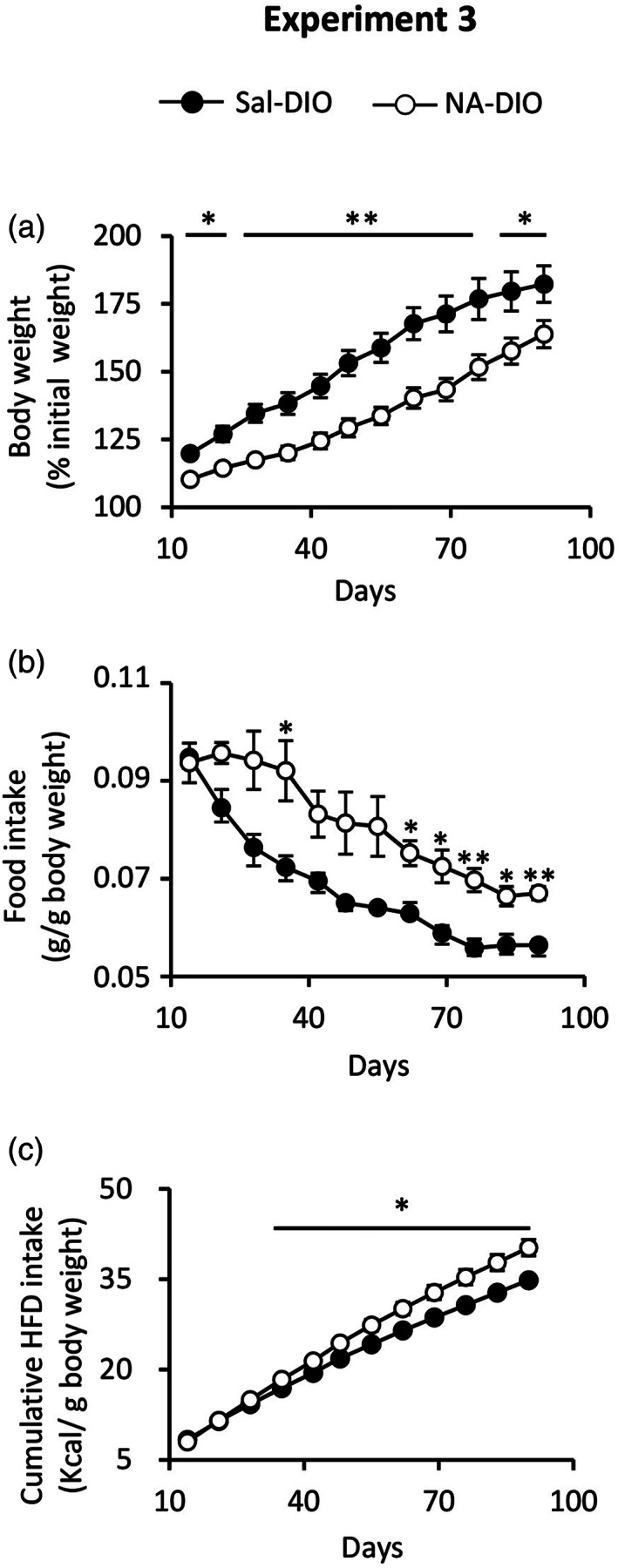
Body weight and food intake of mice who suffered an acute NA‐induced inflammation and later underwent diet induce obesity. One month after an ICV injection of NA or saline, the mice were fed a HFD for 3 months to induce obesity (Experiment 3). (a) Body weight and (b, c) food intake, were recorded weekly from HFD administration. (a) Progression of the body weight, expressed as the percentage of the initial weight (at the beginning of the HFD). Soon after HFD administration NA‐treated mice (NA‐DIO) present a lower weight than control mice (Sal‐DIO), a difference that increases with time. (b) Food intake (relative to body weight) recorded from the beginning of the HFD administration. In spite of gaining less weight, mice treated with NA ate significantly more than saline‐treated mice. (c) The cumulative intake of HFD (expressed as caloric intake relative to body weight) was higher in NA‐treated mice from 1.5 months of HFD on. Data presented are the mean ± *SEM*. Weight is of *n* = 9 mice. Food intake corresponds to that of three mice kept in the same cage, and three cages; therefore, *n* = 3. *p < .05; **p < .01

Our previous results obtained from rats and from isolated microglia suggest a priming state of microglial cells after an inflammatory process, which was provoked by the ICV injection of NA several months before. Thus, it seems that the priming state could be maintained in the long term. In addition, as we had focused on the consequences of the NA‐treatment on the hypothalamus, evidences arose that microglia residing in this brain region might be under such priming state. Rats treated with NA and exposed to a nutritional challenge (a HFD) seem to normally regulate food intake and weight gain (Figure 2). However, a slight difference (although not significant) could be observed in the weight gain curves of NA versus saline treated rats subjected to the HFD, specifically at the end of the HFD period (Figure 2a). This observation prompted us to investigate the possibility that NA‐treatment could provoke long‐term hypothalamic alterations which would jeopardize body weight regulation in the face of a more severe nutritional challenge. Thus, mice were ICV injected with NA or saline, and allowed to recover from inflammation during 1 month. Then, all them were switched to a HFD, which was maintained for 3 months to induce obesity (DIO mice), and weight was monitored weekly from this moment onwards. Results are presented as % of the initial weight, considering this the weight at the moment of switching to the HFD. The mean body weight at this moment (1 month after ICV injection and right before HFD) was 27.9 ± 0.6 g for saline‐injected mice and 26.7 ± 0.7 g for NA‐injected mice. Although the difference in mean body weight was not statistically significant we know, from this and other experiments, that animals treated with NA tended to have a slightly reduced weight compared with saline‐injected animals (what we had previously considered a consequence of the transient sickness that occurs after NA‐induced inflammation). Once switched to the HFD, weight gain (expressed as % of the initial weight) of animals treated with NA (NA‐DIO mice) was slower than that of mice injected with saline (Sal‐DIO mice; Figure 10a). However, in spite of gaining less weight, mice pretreated with NA ate more (g of diet per g body weight) than control mice (Figure 10b). Soon after switching to the HFD, control (Sal‐DIO) mice rapidly reduced food intake to adjust the daily amount of calories ingested to the higher caloric content of the new diet. However, NA‐treated mice maintained the amount of food ingested for a longer period (about 1 month) and therefore had a higher caloric intake than control mice. Still, this did not translate in an increased weight gain. Food intake calculated as cumulative caloric intake relative to body weight was also higher in NA‐treated mice from about 40 days of HFD feeding onwards (Figure 10c).

These results indicate that a past inflammation, in particular that induced by NA, produces long lasting alterations that impair the ability of regulating food intake and body weight when the animals are exposed to a nutritional challenge. We next searched for signs of such alterations in the hypothalamus.

### 3.7 A past event of NA‐induced inflammation alters the hypothalamic adjustment to a diet‐induced obesity

**FIGURE 11:**
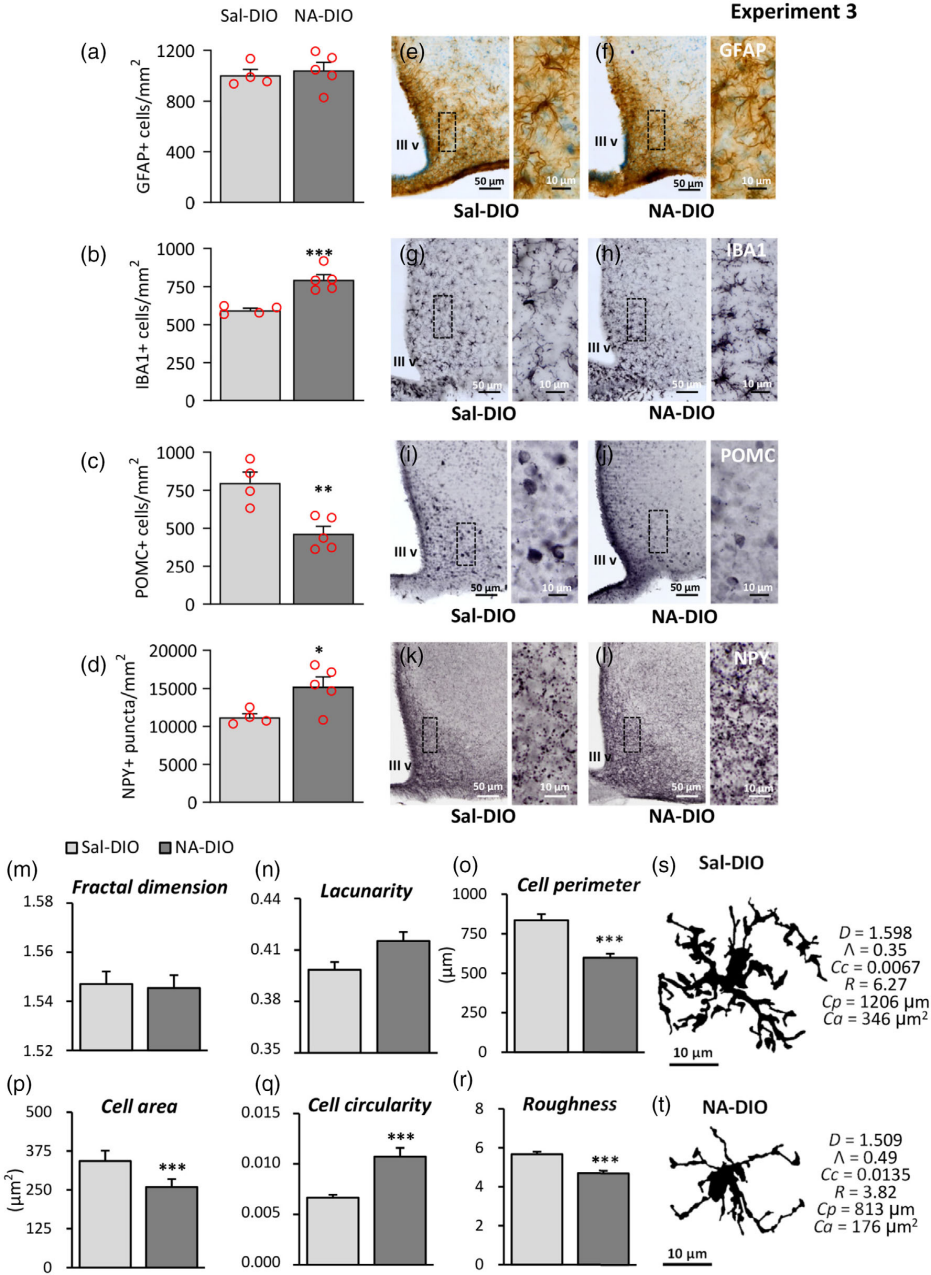
Histological features investigated in the arcuate nucleus of DIO mice that suffered NA‐induced neuroinflammation 3 months before. One month after an ICV injection of NA or saline, the mice were fed a HFD for 3 months to induce obesity (Experiment 3). Sections of hypothalamus including the arcuate nucleus were immunostained for GFAP, IBA1, POMC, and NPY. (a–c) GFAP, IBA1, and POMC‐positive cell counts, respectively, in the arcuate nucleus. (d) NPY‐labeled puncta in the arcuate nucleus. The bars represent mean ± *SEM* of *n* = 4–5 animals per group. To perform morphological characterization of microglial cells, brain sections were immunostained for IBA1. Images of IBA1‐positive microglia located in the arcuate nucleus were processed to obtain a binary image of the cell's profile. These processed images were used to measure morphometric parameters: (m) fractal dimension, (n) lacunarity, (o) cell perimeter, (p) cell area, (q) cell circularity, and (r) roughness. The graphs present the mean ± *SEM* of these parameters measured in *n* = 50 cells from each experimental group. (s, t) Images of representative arcuate nucleus microglial cells from saline‐injected (s) and NA‐treated (t) DIO mice. For these particular cells, the values of the morphological parameters presented in the graphs are shown below their profiles. *D*, fractal dimension; *Λ*, lacunarity; *cc*, cell circularity; *R*: Roughness; *Cp*: Cell perimeter; *ca*: Cell area. *p < .05; **p < .01; ***p < .001

Brain sections from DIO mice pretreated with NA or saline‐injected months before were immunostained to explore the hypothalamus, particularly the ARC containing orexigenic (AgRP/NPY expressing) and anorexigenic (POMC/CART expressing) neurons. First, astrogliosis was examined by GFAP staining, but no difference was found in the number of GFAP‐positive cells in the ARC between NA‐DIO and saline‐DIO mice (Figure 11a). On the contrary, the number of microglial cells (IBA1‐positive) was significantly higher in NA‐DIO mice (Figure 11b). Not only the number but also the morphology of these microglial cells was different, as revealed by the quantification of morphological parameters (Figure 11m–t; Figure S3). While some parameters like *fractal dimension* (Figure 11m) or *lacunarity* (Figure 11n) were similar in microglial cells from both experimental groups, others like *cell perimeter* (Figure 11o), *cell area* (Figure 11p), *cell circularity* (Figure 11q), and *roughness* (Figure 11r) were significantly different in microglial cells of NA‐DIO mice compared with those sampled from Sal‐DIO mice. The morphological changes measured in NA‐DIO microglia were indicative of smaller and more compact cells with fewer ramifications, suggestive of a more activated state. In fact, visual inspection of two sampled cells (Figure 11s,t) evidenced this morphological difference. Therefore, under DIO conditions microglial cells from mice pretreated with NA seemed to be more activated than those from saline‐injected controls.

On the other hand, cell counts of POMC‐positive cells in the ARC showed a reduction of anorexigenic POMC cells in NA‐DIO mice (Figure 11c). Conversely, NPY staining revealed a higher number of NPY‐positive puncta in the ARC of NA‐DIO mice (Figure 11d). Thus, NA‐injection was provoking also an unbalance in the neuronal populations regulating the energy balance located in ARC, at least under DIO conditions.

**FIGURE 12:**
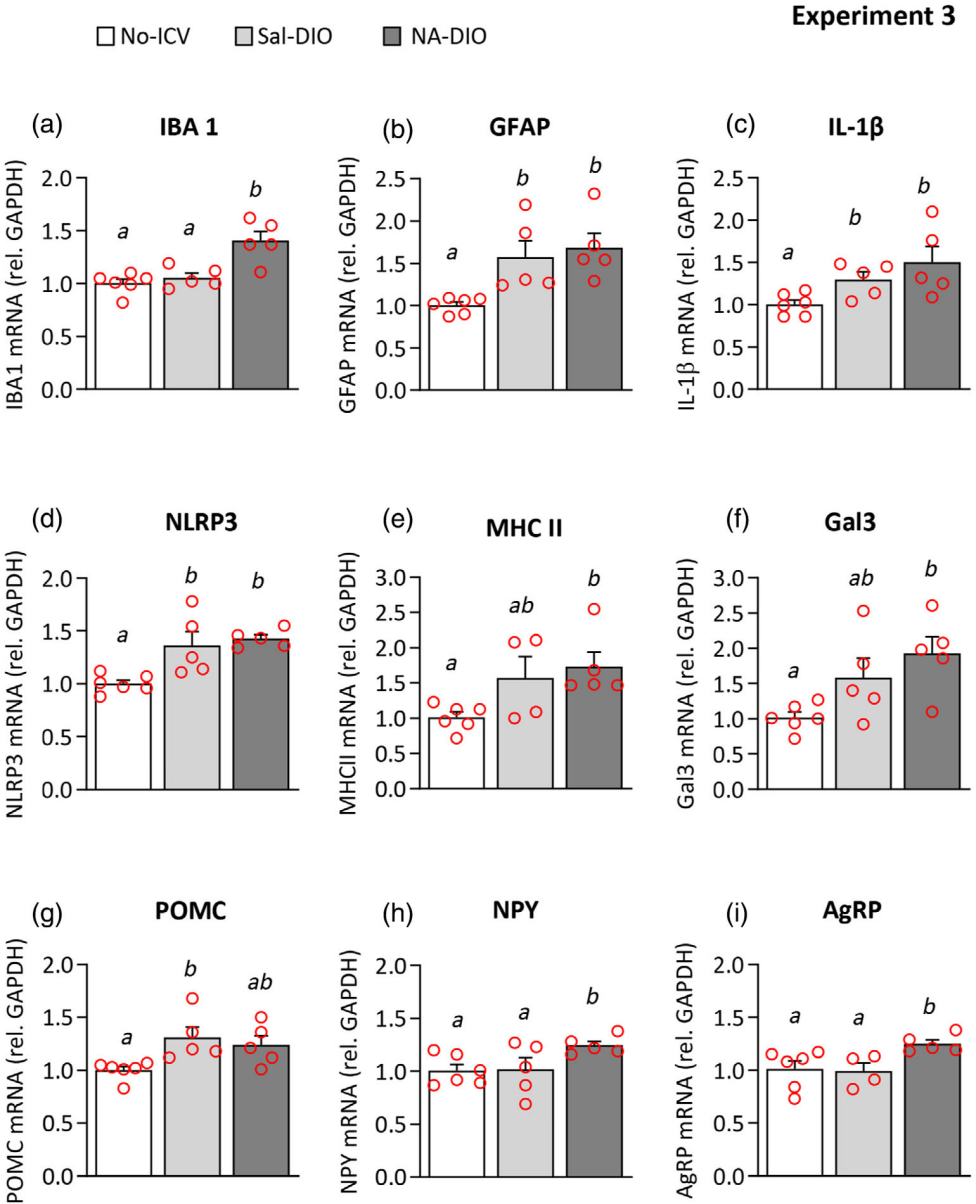
Quantification of gene expression in the hypothalamus of DIO mice that previously suffered NA‐induced neuroinflammation. One month after receiving an ICV injection of NA (NA‐DIO) or saline (Sal‐DIO), mice were fed a HFD for 3 months to induce obesity (Experiment 3). The hypothalamus was then dissected out and processed for qPCR. Samples from non‐injected mice (No‐ICV) were also used as a reference of basal gene expression. (a–i) The graphs show mRNA levels expressed relative to the housekeeping gene GAPDH. The bars in the histograms represent the mean ± *SEM* of *n* = 5–6 animals per group, and the letters *a–b* on top of the bars indicate the absence (if the same letter appears) or existence (if different letters appear) of a statistical difference (*p* < .05)

Besides, gene expression analysis was performed in whole hypothalami obtained from NA‐DIO and Sal‐DIO mice. Hypothalami from age‐matched, non‐injected normal mice (fed with regular diet) were used as well as a reference of basal gene expression. The expression of IBA1 (Figure 12a), NPY (Figure 12h), and AgRP (Figure 12i) was increased in NA‐DIO mice compared with Sal‐DIO mice, while the expression of other genes (GFAP, IL‐1β, NLRP3, MHC II, Gal3, POMC; Figure 12b–g, respectively) remained unchanged. These results point out that a past NA‐inflammatory event slightly alters the gene expression pattern (IBA1, NPY, AgRP) in the hypothalamus under DIO conditions, thus supporting our previous results. Interestingly, the inclusion of samples from No‐ICV mice allowed to observe that DIO enhanced the expression of genes related to inflammation (GFAP, IL‐1β, NLRP3, MHC II, Gal3, Figure 12b–f, respectively) irrespective of the prior ICV treatment. Although DIO is most likely responsible for such increases in gene expression, we cannot rule out the possibility that the ICV procedure itself (carried out several months before in DIO mice, but not in No‐ICV mice) might contribute as well.

Regarding POMC gene, its expression was increased in Sal‐DIO mice compared with No‐ICV mice. However, such increase was slightly reverted (although not statistically significant) in NA‐DIO mice (Figure 12g), what is in accordance with the reduced number of POMC‐positive cells in the ARC of NA‐DIO mice compared with Sal‐DIO mice.

Therefore, body weight and food intake disturbances observed in NA‐DIO mice were associated with hypothalamic alterations, at least in the ARC, involving microglia as well as neurons controlling feeding and weight balance. The inflammatory event provoked by the ICV injection of NA has long‐term repercussions in the hypothalamus. Although those consequences are quite subtle, they may emerge in a DIO situation.

## 4 DISCUSSION

### 4.1 Hypothalamic microglia become primed after neuraminidase‐induced neuroinflammation, a state that persist over time

In recent past years the primed state of microglial cells has emerged as a novel cellular status, which has gained interest after evidences pointing that it might be underneath neurodegeneration, cognitive impairment and behavioral disturbances (De Sousa et al., 2021; Frank et al., 2020; Lisboa et al., 2016; Norden et al., 2015; Perry & Holmes, 2014; Wendeln et al., 2018; Yirmiya et al., 2015). Here the attention was focused on the potential impact of primed microglia on basic hypothalamic functions like energy balance regulation. Also, we aimed at the possibility that such state could remain for a long period of time, therefore affecting or interacting with situations (e.g., trauma, infections, or overfeeding) arising later in life. A model of acute neuroinflammation previously devised by our research group was used to address these issues. It is based on the ICV administration of the enzyme neuraminidase (NA), which is part of bacteria and viruses such as influenza virus. NA is administered in the lateral ventricle, from where it flows to the third and fourth ventricles. NA provokes an inflammatory process with many of the classical features of neuroinflammation, which has been described elsewhere (Fernandez‐Arjona et al., 2017; Fernandez‐Arjona et al., 2019a; Granados‐Durán et al., 2015; Granados‐Durán et al., 2016; Grondona et al., 1996). Most inflammation markers peak during the first week, and 2 weeks after the injection of NA most signs of inflammation have disappeared (Granados‐Durán et al., 2015). The inflammatory reaction is more evident at the injected lateral ventricle; however, as NA flows caudally, other periventricular areas are also mildly affected. Among them, evidences of microglial activation can be observed in the hypothalamus (Fernandez‐Arjona et al., 2017; Fernandez‐Arjona et al., 2019b). The mediobasal portion of the hypothalamus was chosen to investigate the priming of resident microglial cells after NA‐induced inflammation, as this region is critical in, among various functions, the regulation of food intake, body weight, and energy balance (Hill, 2010). In the hours following NA‐injection, hypothalamic microglia located close to the ventricular surface show signs of activation, including IL‐1β expression and morphological changes consistent with activation (Fernandez‐Arjona et al., 2017; Fernandez‐Arjona et al., 2019a, 2019b). Here we demonstrate that those changes in microglia are mostly reversible: 3 months after the ICV injection no morphological differences were found in hypothalamic microglia sampled from NA‐treated rats and saline‐injected controls, nor did they express IL‐1β (Experiment 1, Figures 4 and 7). However, NA provoked alterations in the hypothalamus that remained in the long term, namely a population of MHC II‐positive cells located in the basal hypothalamus which seems to be microglial cells (Figures 3 and 4), and the upregulation of certain inflammation related genes such as the inflammasome protein NLRP3 and the endogenous lectin Gal3 (Figure 7). Interestingly, and in accordance with immunostaining results, IL‐1β gene expression was similar in NA and saline injected animals 3 months after the ICV injection, and no signs of gliosis were found (same IBA1 and GFAP cell counts in the ARC), indicating that inflammation is largely solved over time. Thus, these results point that microglia primed state is compatible with a non‐inflamed scenario. The population of MHC II‐positive cells found in the basal hypothalamus of NA‐treated rats was not accompanied by an increased expression of MHC II by qPCR (measured in whole hypothalamus); however, a hint of an increase was observed. This is probably due to the small proportion that these cells represent in the whole hypothalamic tissue that was used for RNA extraction and qPCR (Experiment 1). In a subsequent experiment performed in mice (Experiment 2), microglia were isolated to evaluate their primed state. In this case, MHC II gene expression was upregulated in microglia isolated from NA‐injected mice, as well as the endogenous lectin Gal3 (Experiment 2, Figure 8), but not so the inflammasome protein NLRP3. It should be noted that in this experiment microglia were isolated from the whole brain, so cells from additional regions other than the hypothalamus could contribute to the observed results. In fact, we hypothesize that, in the NA‐inflammation model, only a modest fraction of microglial cells might get primed; therefore, any assessment technique (i.e., qPCR in whole hypothalamus extracts, microglia isolation from the whole brain) that includes all microglial cells will underscore the features of primed microglia. In this same line, our histology results (cell counts and microglia morphological analysis) obtained specifically from the ARC gain relevance. These facts should be taken into account in assessing the results presented here.

Therefore, these results indicate that after NA‐induced inflammation mild changes in microglial cells persist over time, and lasts as long as, at least, up to 3 months after the inflammatory stimulus. Although this work focuses on priming of microglial cells, the possibility that astrocytes may as well result primed after NA‐induced inflammation should be taken into account (Hennessy et al., 2015; Muccigrosso et al., 2016).

A relevant question regarding immune priming in the nervous system is the timespan this state may endure (Neher & Cunningham, 2019). Most studies focus on relatively short time frames, usually within 1 month from the priming stimulus (Frank et al., 2020; Muccigrosso et al., 2016; Purisai et al., 2007; Weber et al., 2015). Our results show that microglial priming is a long lasting state that remains at least up to 3 months. Other authors have also reported the enduring character of microglial priming. In rodents, chronic activation of microglia has been observed months or even 1 year after traumatic brain injury (Holmin & Mathiesen, 1999; Loane et al., 2014) or sepsis (Trzeciak et al., 2019); however, these authors describe a chronic activation state of microglia, rather than a primed state. Conversely, our results do not support a chronic inflammatory situation. These differences could be related to the nature and/or severity of the inflammatory stimulus: as previously mentioned, NA‐induced inflammation has been regarded as an acute process mostly limited to periventricular areas (Granados‐Durán et al., 2015). Interestingly, Wendeln and colleagues reported immune memory in the brain after a peripheral inflammatory stimulation, which lasted for at least 6 months and, more importantly, determined the outcome of neurological diseases (Wendeln et al., 2018). Therefore, after an initial priming stimulus, the detrimental consequences of any subsequent inflammatory stimulus may arise even months after the initial priming stimulus.

### 4.2 Enhanced response of primed hypothalamic microglia to HFD

Primed microglia are characterized by an exacerbated response to inflammatory stimuli. To induce such response in hypothalamic microglia, NA and saline‐injected rats were fed a HFD for 10 days, which is known to provoke mild hypothalamic inflammation (De Souza et al., 2005; Thaler et al., 2012). After 10 days of HFD NA‐treated rats gained similar weight than saline controls. However, a hint of a slightly diminished weight gain in NA‐rats by the last days was observed (Experiment 1, Figure 2). Food intake was the same in both experimental groups, with a progressive reduction in HFD consumption, evidencing the proper functioning of the regulatory mechanism aimed to adjust the net caloric intake. As previously reported (Thaler et al., 2012), HFD provoked mild hypothalamic inflammation, that was revealed by increased numbers of astrocytes and microglia in the ARC (Figure 3). In addition, microglial morphological parameters pointed out microglial activation (reduced *cell perimeter* and *fractal dimension*) upon HFD feeding (Figure 6). However, it is somewhat puzzling that, conversely to results reported by other authors (De Souza et al., 2005; Thaler et al., 2012), HFD did not significantly alter the expression of inflammation related genes in the hypothalamus, as demonstrated by comparing saline injected rats fed either chow or HFD (Figure 7). Similarly, to our results, unaltered gene expression in the hypothalamus after 1 week HFD has been also reported (Valdearcos et al., 2014). The impact of HFD on hypothalamic gene expression seems to vary among experiments, probably depending on the duration of the exposure to the diet, the animal species or strain, and the method used for the quantification of gene expression.

Interestingly, signs of a somewhat increased response (although not excessive) to the HFD could be observed in the hypothalamus of NA‐treated rats, which presented a slightly higher expression of IBA1 (Figure 7) and increased numbers of MHC II‐positive cells (Figure 3) than the corresponding NA‐treated chow‐fed controls. The morphological analysis of microglial cells sampled from the ARC further confirmed a heightened response to the HFD in microglia from NA‐exposed rats (Figure 6). Moreover, microglia isolated from the whole brain of mice and in vitro stimulated with LPS and/or PA presented a similar response, with a higher IL‐1β expression in cells isolated from NA‐injected mice (Experiment 2, Figure 9). These results point that a prior acute inflammatory event, in this case induced by the ICV injection of NA 3 months before, provokes subtle changes in the hypothalamus that persist over time, affecting specifically microglial cells, which present signs of priming. Among those enduring alterations, a population of MHC II‐expressing microglial cells located in the basal hypothalamus, which is absent in naïve animals, stands out, as well as a higher expression of Gal3.

A variety of experimental designs have been employed to investigate innate immune memory in the brain or, more specifically, microglial priming. These works combine two inflammatory stimuli: a primary stimulus, which induces priming, and a secondary stimulus, which triggers an exacerbated immune response in the brain. Primary stimuli range from natural (aging) or induced (different animal models) neurodegeneration, traumatic brain injury, central or peripheral LPS injections, or even sepsis (reviewed in Neher & Cunningham, 2019). Secondary stimuli are less variable, the most common being a peripheral LPS injection, which may provoke a robust or a milder immune response depending on the dose (Neher & Cunningham, 2019). Here we used a novel‐priming stimulus, namely the ICV administration of NA, because this enzyme is part of several brain pathogens, and also because it provokes an acute inflammation largely studied by our group (Fernandez‐Arjona et al., 2019a, 2019b; Granados‐Durán et al., 2015; Granados‐Durán et al., 2016). Once injected, NA spreads in the cerebrospinal fluid, and is able to cross the ependymal layer and reach several microns beneath it (Granados‐Durán et al., 2015). NA has been shown to directly activate microglial cells, acting mainly through TLR4 receptors (Fernandez‐Arjona et al., 2019a). The importance of microglial TLR4 signaling for acquiring the primed state is unknown. In addition to the possible direct role of NA in inducing microglial priming, the involvement of other inflammatory mediators produced during NA‐induced inflammation should be considered. In fact, this latter possibility gains prominence in the case of microglia located in the basal hypothalamus as, conversely to other periventricular areas, this region is protected behind the ventricular barrier established by tanycytes, and therefore most probably secluded to the reach of NA diluted in the cerebrospinal fluid. As secondary stimulus we chose an unusual one, a HFD, which is recognized as a mild inflammatory stimulus to the hypothalamus (Thaler et al., 2012; Valdearcos et al., 2014). Thus, our work provides evidence that nutritional signals associated to particular diets (such as HFD) may amplify the inflammatory response in the previously challenged brain. In the present work, the relatively modest response of primed microglia to this secondary stimulus should take into account the mild nature of the HFD (as reported by other authors) as hypothalamic inflammatory stimulus.

### 4.3 A prominent population of MHC II‐positive microglia arises in the hypothalamus of rats that suffered NA‐induced neuroinflammation

As mentioned before, a noticeable population of MHC II‐positive cells was found in NA‐treated rats in this study. These cells, which were also IBA1‐positive, were mostly located in the median eminence, in the ARC and, to a lesser extent, in subependymal areas of the third ventricle. Besides being associated to NA‐treatment, short (10 days) HFD feeding increased its number. Because they were particularly profuse in the external layer of the median eminence, where blood vessels are abundant, the question arises if they are infiltrated monocytes/macrophages instead of resident microglial cells. None of these cells turned out to be positive with the monocyte marker Ly6c. It is well known that Ly6c‐positive monocytes acutely infiltrate into the brain during inflammation induced by peripheral sepsis (Trzeciak et al., 2019), brain stroke (Hammond et al., 2014; Miro‐Mur et al., 2016), or traumatic brain injury (Hsieh et al., 2014; Morganti et al., 2015). However, infiltrated monocytes transform into microglia/macrophages later on, loose Ly6c expression and start expressing IBA1, acquiring a phenotype similar to microglia (Miro‐Mur et al., 2016). On the other hand, other inflammatory stimuli like HFDs or obesity itself are associated to peripheral immune cell infiltration (Buckman et al., 2014). In fact, specific monocyte markers (such as CD169) allowed to determine the significant contribution of infiltrated cells to the microgliosis observed in the mediobasal hypothalamus after feeding mice with HFD during 4 weeks (Valdearcos et al., 2017), but not so much at shorter feeding times (as is our case). Therefore, in the present work, monocytes infiltrated during NA‐induced inflammation months before could have differentiated into IBA1‐expressing macrophage/microglia‐like cells. The question of the origin of hypothalamic MHC II/IBA1‐positive cells observed here remains therefore open, and the possibility that they derive from infiltrated monocytes should be taken into consideration.

On the other hand, MHC II/IBA1‐positive cells were also found in other locations, particularly nearby the site of the ICV injection, in the cortex and corpus callosum. Conversely to those found in the basal hypothalamus, these cells appeared mostly with a surveillant ramified morphology, and a fainter MHC II staining. We speculate that these, rather than infiltrated cells, may be resident microglia, activated by the surgical procedure of the injection. Overexpression of MHC II by microglial cells has been described in animals long after brain injury (Holmin & Mathiesen, 1999; Muccigrosso et al., 2016). In fact, the injection procedure itself is a traumatic event that triggers inflammation, which could also contribute to the priming of microglia. This fact is evident in our results from whole brain isolated and in vitro stimulated microglia (Experiment 2), where microglial IL‐1β response tended to be higher when they were isolated from ICV saline‐injected mice than when obtained from naïve (non‐injected) mice (Figure 9). MHC II expression by hippocampal microglial cells also occurs progressively during aging and independently of brain injury (VanGuilder et al., 2011), thus supporting the possibility that MHC II‐positive cells found in cortex/corpus callosum are actually modified (primed?) resident microglial cells. On the contrary, as the basal hypothalamus is distant from the injection site it is improbable that MHC II/IBA1‐positive cells found in this location are a result of surgical injury. Their potential peripheral origin, therefore, needs to be considered. Animal models where monocyte infiltration is widely limited (such as CCR2 knockout mice) and monocyte‐specific makers (like CD169) would be useful to address this question. Furthermore, the impact of infiltrated cells on brain functions should be investigated, as several authors have reported a detrimental outcome (Hammond et al., 2014; Hsieh et al., 2014; Trzeciak et al., 2019; Valdearcos et al., 2017).

### 4.4 MHCII, galectin3 and NLRP3 inflammasome are overexpressed in primed microglia

A precise phenotype of primed microglia has not been defined yet. MHC II has been reported as a marker of primed microglia (Ling et al., 2004; Muccigrosso et al., 2016) which, in non‐injured rats, is expressed by an increasing proportion of hippocampal microglial cells during aging progression (VanGuilder et al., 2011). Although MHC II is sometimes regarded as a marker of activated microglia (Holmin & Mathiesen, 1999; Loane et al., 2014; Trzeciak et al., 2019) this does not seem to be the case here, as we observed MHC II/IBA1‐positive cells with morphologies proper of activated as well as typical of resting cells. Also, none of them expressed the pro‐inflammatory cytokine IL‐1β, indicating that, if activated cells, their activation state is not the classical pro‐inflammatory one. Furthermore, MHC II overexpression has also been reported in a subset of microglial cells at late stages of neurodegeneration (Mathys et al., 2017). This highlights the variety of states that microglial cells can engage, or even the fact that different microglial subtypes do actually exist (Stratoulias et al., 2019). Our results point that MHC II might be considered a marker of primed, but not necessarily activated, microglia. Future research will clarify this issue, as well as the yet unknown role of this antigen presenting molecule expressed by microglia in a context where no foreign antigens are present nor inflammation is currently undergoing. In this sense, it has been suggested that in proteinopathies, where misfolded or toxic proteins accumulate in the nervous parenchyma, microglia engage in self‐antigen presentation (e.g., amyloid‐β or phosphorylated Tau) through MHC I/II, what favors the restorative profile of microglial cells (reviewed in Das & Chinnathambi, 2019). Also, it is suggestive the novel notion that in scenarios such as inflammation, where large amounts of auto‐antigens are being processed by resident antigen presenting cells (like microglia in the nervous tissue), these cells may engage a “paralyzed” phenotype, characterized by the overexpression of MHC II in their surface (Sadegh‐Nasseri & Kim, 2015).

The priming of the inflammasome NLRP3 is an initial step of its activation that involves the production of a threshold amount of NLRP3 protein (Swanson et al., 2019). In animals who suffered either acute or chronic stress, NLRP3 inflammasome priming was shown to be essential for microglia to shift to a primed state (Alcocer‐Gómez et al., 2016; Feng et al., 2019; Weber et al., 2015), a state that lasted at least up to 8 days in vivo and even up to 28 days in isolated microglia (Frank et al., 2020). Our results demonstrate that a different type of inflammatory stimulus, as is the ICV administration of NA, is also able to induce NLRP3 overexpression (inflammasome priming) in the rat hypothalamus (Experiment 1). Notably, NLRP3 priming in the hypothalamus persisted even 3 months after the application of the priming stimulus, even though other typical inflammatory markers (IL‐1β) were not increased. However, NLRP3 priming was not observed in isolated microglia 3 months after treating the mice with NA (Experiment 2); here again, an underrepresented primed population of microglial cells may mask primed microglia features. Thus, the priming of NLRP3 inflammasome (which may be triggered by different inflammatory stimuli including NA) can persist for a long period of time (up to 3 months according to our results), and could be a key event for microglial priming.

The expression of several genes related to inflammation quantified 3 months after an acute inflammatory stimulus showed no differences compared with control animals (Experiment 1, Figure 7; Experiment 2, Figure 8), indicating that the inflammatory status in our experimental paradigm (i.e., a past NA‐induced inflammation) is mild. Yet, interestingly the expression level of the lectin Gal3 remained increased several months after NA‐injection. Such increased expression of Gal3 was observed in isolated microglial cells (Experiment 2, Figure 8), although other cell types (e.g., astrocytes) could also be affected (Sirko et al., 2015). These results point that Gal3 might be proposed as a marker of microglial priming. Besides, experiments with isolated microglia revealed that Gal3 expression increased not only with the injection of NA, but also by the surgical procedure itself (Experiment 2, Figure 8), indicating the diverse nature of stimuli contributing to Gal3 overexpression. Gal3 is an inflammatory modulator expressed by neurons, astrocytes and, most notably, activated microglia, which has been assigned a role as an alarmin that aids spreading inflammation to other brain areas (Burguillos et al., 2015; Yip et al., 2017). In a model of head trauma Gal3 was reported to promote inflammation and neurodegeneration, acting as a ligand for the receptor TLR4 (Burguillos et al., 2015; Yip et al., 2017). However, in a stroke model Gal3 promoted a shift of microglia to a restorative phenotype in later stages of inflammation (Rahimian et al., 2019). In addition, Gal3 is overexpressed in microglia in late stages of neurodegeneration (Mathys et al., 2017). Although the association of Gal3 to neuroinflammation is beyond question, its specific roles seem to depend on the type of inflammatory process and the time frame of its action. Undoubtedly Gal3 deserves further investigations.

Another indication of the primed state of microglia after a past NA‐induced inflammation arises from in vitro stimulation experiments, which showed an enhanced IL‐1β response to LPS in microglia isolated from NA‐treated mice (Experiment 2, Figure 9) (although a milder response than we had expected, maybe because of the low fraction of primed microglia, as previously hypothesized). On the other hand, these experiments also reveal the previously exposed contribution of the ICV procedure (including both, tissue injury and potential bacterial contamination) related inflammation to microglial priming (by comparing Sham‐ICV to No‐ICV groups), a fact that needs to be taken into consideration when elucidating future experiments where the ICV administration route is used. Of note, in our experiments palmitic acid (PA) was not able to stimulate microglia; in spite of a trend to induce the overexpression of IL‐β, the expression levels of this cytokine were not statistically different from those in control cultures. This result differs from works by other authors, which report that PA is able to stimulate microglial cells (Valdearcos et al., 2014; Yanguas‐Casas et al., 2018). However, PA did not affect basal microglial activity (migration and phagocytosis), but only that resulting from interferon‐γ stimulation (Yanguas‐Casas et al., 2018). Thus, PA actions on microglia may be relevant only in specific situations, what could explain the absence of response in our in vitro experiments.

### 4.5 Alterations in the anorexigenic hypothalamic circuitry, along with body weight and food intake dysregulation, in DIO mice long after acute neuroinflammation

The results obtained from Experiments 1 and 2 lead us to conclude that NA‐induced inflammation was able to provoke subtle but long‐lasting effects in the brain, particularly in the hypothalamus, and more precisely in the microglial population located in the basal hypothalamus including the ARC. Moreover, upon a second inflammatory stimulus, in this case a mild one as is a transient exposure to a HFD, these microglial cells executed an enhanced response, indicative of a bias to a primed state. Also, no signs of altered regulation of body weight or food intake were observed. However, we wondered if a stronger inflammatory challenge to the hypothalamus would be able to unbalance body weight and food intake regulation. Therefore, by feeding a HFD for a longer period, we generated obesity to mice who previously suffered NA‐provoked inflammation (Experiment 3).

Upon switching to HFD, mice that had received an ICV injection of NA took longer to reduce their food intake, and therefore their caloric intake was higher than that of control mice ICV‐injected with saline. In spite of maintaining a higher food intake over time, mice treated with NA gained less weight than control mice (Experiment 3, Figure 10), what could be indicative of a higher energy expenditure. Besides, at the basal hypothalamus, these mice presented more microglial cells in ARC (Figure 11b), which also had morphological features skewed to an activated state (Figure 11m–t). IBA1 gene expression was also higher (Figure 12a). Furthermore, additional alterations were found in this brain region, such as a reduced population of POMC‐positive cells and increased number of NPY puncta (Figure 11c,d). As POMC neurons are recognized as part of the anorexigenic hypothalamic circuit (Dietrich & Horvath, 2013; Morton et al., 2006; Morton et al., 2014), a reduction of its population could explain the increased food intake observed in NA‐treated mice under HFD. In addition, the increased number of NPY‐puncta in the vicinity of POMC cells indicates an enhanced inhibitory input of AgRP/NPY neurons (the orexigenic neuronal population of the ARC) over this POMC population (Csiffary et al., 1990; Horvath et al., 1992). In the same sense, NPY and AgRP gene expression were slightly increased in NA‐treated mice (Figure 12h‐i). Thus, in DIO mice the previous NA‐induced inflammation provokes a downregulation/inhibition of the anorexigenic POMC neuronal population in the ARC.

Interestingly, these hypothalamic POMC neurons have been reported to be particularly sensitive to nutritional overloads. Thus, Moraes et al. observed that HFDs induce apoptosis of hypothalamic POMC neurons (Moraes et al., 2009). In addition, chronic HFD provoked a reduction in the number of hypothalamic POMC neurons which was associated to weight gain and obesity (Thaler et al., 2012; Yip et al., 2017). An induction of Hsp72 in ARC neurons during HFD feeding indicates the activation of a neuroprotective response in these cells (Thaler et al., 2012). In fact, POMC neurons are considered to protect against obesity, so their loss compromises body weight regulation. Mitochondrial stress in POMC neurons has been suggested as a possible mechanism underlying this neuronal loss (Yip et al., 2017), as well as IKKb NF‐kB activation and ER stress in hypothalamic neurons induced by overnutrition (Zhang et al., 2008). On the other hand, a reorganization of the synaptic input onto POMC neurons exposed to HFD has been described as well (Horvath et al., 2010). As it is well accepted that HFD induces hypothalamic inflammation even before the development of obesity (Thaler et al., 2012), inflammation could be the final cause of hypothalamic POMC neuronal loss/inhibition. Any causes that may aggravate hypothalamic inflammation (such as the priming of microglial cells induced by NA) would worsen such neuronal loss. On the other hand, we have considered that a direct action of NA on the POMC neuronal population might account for their loss in the hours following the ICV injection. However, this seems quite unlikely because, as previously explained, the ARC is precluded from cerebrospinal fluid born molecules by the barrier established by tanycytes at the mediobasal hypothalamus.

Additional evidences demonstrate the participation of hypothalamic glial cells, particularly microglia, on neuronal survival and/or synaptic remodeling within the basal hypothalamus. Activation of hypothalamic microglia using a TLR2 agonist resulted in increased activity of POMC neurons and altered synaptic input onto these cells (Jin et al., 2016). In a hypercaloric environment, a sustained activation of microglial cells and increased TNFα secretion provoked POMC cells loss (Yip et al., 2017). The pattern recognition receptor TLR4 has been shown to be the primary target of saturated fatty acids (Milanski et al., 2009) which abound in HFDs and accumulate in the hypothalamus of HFD fed animals (Valdearcos et al., 2014). Microglial cells, which rapidly reacts to HFDs, express both TLR2 and TLR4 receptors, and are proposed to act as hypothalamic sensors of saturated fatty acids. Furthermore, they seem to orchestrate an inflammatory process that, in the context of HFD, modifies neuronal activity in the basal hypothalamus (Valdearcos et al., 2014; Valdearcos et al., 2017). Depletion of microglia using a specific drug lessened the obesogenic effects of HFD (Valdearcos et al., 2014), while in healthy rats, depletion of microglia by a genetic strategy provoked anorexia and weight loss (De Luca et al., 2020). Also, LPS (an agonist of TLR4) applied to brain slices resulted in changes in the firing activity of POMC and AgRP/NPY neurons, and microglia inhibition with minocycline altered feeding in mice (Reis et al., 2015). Thus, hypothalamic microglia are a crucial player in the regulation of feeding and energy balance, particularly in the context of a nutritional overload as is a HFD (Valdearcos, Myers, & Koliwad, 2019).

Hypothalamic glial cells (including astrocytes, microglia, and tanycytes) are gaining prominence in their contribution to hypothalamic plasticity, supporting the remodeling of neuronal circuits, tuning the sensitivity of neurons to nutritional cues, or adjusting the access to the brain of those peripheral cues (García‐Cáceres et al., 2019; Valdearcos et al., 2019). The contribution of glial cells to disturbances affecting the different hypothalamic–pituitary axes undoubtedly deserves further investigations.

Primed microglia are characterized by a higher sensitivity and an exacerbated response to inflammatory stimuli. During aging, microglia progressively acquire this primed condition, what contributes to neurodegeneration and cognitive impairment (Norden et al., 2015; Norden & Godbout, 2013; Perry & Holmes, 2014; Perry & Teeling, 2013; Wynne et al., 2009). Age‐primed microglia, and particularly that located in the amygdala, seems to be particularly sensitive to the inflammatory stimulus that a HFD represents (Spencer et al., 2019). Similarly, because saturated fatty acids build up in the basal hypothalamus (Valdearcos et al., 2014), primed microglia in this region may also be particularly vulnerable to HFDs. Although priming of hypothalamic microglia has not been previously reported, it may have a significant impact in the regulation of multiple processes, for example, energy balance and metabolism. Interestingly, traumatic brain injury in mice causes hypothalamic alterations such as hypertrophied astrocytes, increased permeability of the blood–brain barrier, and tanycyte dysfunction (Osterstock et al., 2014), as well as long‐term astrocytosis in the hypothalamo‐pituitary axis of rats (Kasturi & Stein, 2009), all of which are compatible with the consequences of inflammation. Furthermore, in humans hypothalamo‐pituitary dysfunctions and endocrine deficiencies following traumatic brain injury are well described (Schneider et al., 2007). Therefore, traumatic brain injury, which is accompanied by an inflammatory process, may result in long‐lasting alterations in the hypothalamo‐pituitary axis, despite its anatomical distance to the injured site. Likewise, NA‐induced inflammation could have similar outcomes. We hypothesize that priming of microglia after NA‐induced inflammation (or after traumatic brain injury as reported in other studies) could be at least partially involved in those hypothalamic deficiencies. Therefore, a priming stimulus, such as traumatic brain injury or NA administered ICV, as is our case, may predispose hypothalamic microglial cells to undergo a neurotoxic response when animals are chronically fed a HFD. In fact, as previously mentioned, primed microglia are more sensitive to HFDs (Spencer et al., 2019).

In addition to an increased food intake, the reduction/suppression of the POMC anorexigenic cell population should also result in a lower energy expenditure and increased body weight (Challis et al., 2004; Elias et al., 1998). However, in our experiments (Experiment 3) NA‐treated mice gained less weight, in spite of eating more (Figure 10). The view that prevails today is that signaling through the melanocortin 4‐receptor (MC4R, the receptor of the POMC‐derived anorexigenic peptide αMSH) regulates both food intake and energy expenditure, as pivotal elements of energy balance regulation, in an inverse manner, that is, when food intake increases energy expenditure decreases, thus leading to body weigh increase (Dietrich & Horvath, 2013; Morton et al., 2014). However, food intake and energy expenditure are regulated by different extra‐hypothalamic MC4R‐expressing neuronal populations, all of them targeted by hypothalamic POMC neurons (which are in fact a heterogeneous population within the hypothalamus). Therefore, a divergent regulation of both functions, food intake and energy expenditure, seems to exist (Balthasar et al., 2005; García‐Cáceres et al., 2019; Koch & Horvath, 2014), which could explain our apparently conflicting results.

### 4.6 Conclusions

Thus, we may conclude that NA‐induced inflammation provokes long‐lasting alterations in the basal hypothalamus, which involve not only microglia but also neurons conforming the energy balance circuitry located in this region. We hypothesized that priming of microglia could be an underlying mechanism. In fact, hypothalamic microglial morphological features indicate an enhanced response of these cells to DIO. Such long‐lasting hypothalamic alterations resulting from a past inflammation may account for a deranged response to any future nutritional and/or inflammatory challenge, as is the obesity that develops with HFDs. The possibility that these overreactive microglia are involved in changing the neuronal populations in charge of regulating feeding and energy balance is a suggestive idea that will require further experiments.

These results evidence that a past neuroinflammatory event, even it being transient, may worsen the outcome of future episodes of inflammation. It is not needed for the secondary inflammatory stimulus to be severe nor related to infections, as is the paradigm of DIO used here. The basal hypothalamus, a pivotal hub for the regulation of autonomous functions, was shown here to be particularly sensitive to inflammation‐induced long‐lasting alterations. Besides the resulting disturbed regulation of the energy balance, the impact of such alterations in other autonomous or neuroendocrine reflexes should be studied as well.

## AUTHOR CONTRIBUTIONS

Conceptualization, María Dolores López‐Ávalos; Jesús M. Grondona; Data curation, María Dolores López‐Ávalos, Jesús M. Grondona; Formal analysis, Mar Fernández‐Arjona, Ana León‐Rodríguez; Supervision, María Dolores López‐Ávalos, Jesús M. Grondona; Writing original draft, María Dolores López‐Ávalos, Mar Fernández‐Arjona; Statistical analysis, Mar Fernández‐Arjona; Ana León‐Rodríguez; Writing review and editing, María Dolores López‐Ávalos, Jesús M. Grondona.

## CONFLICT OF INTEREST

The authors declare that there is no conflict of interest.

## Supporting information


**Appendix S1:** Supporting Information.

## Data Availability

Data sharing is not applicable to this article as no new data were created or analyzed in this study.
