# Supplementary material for: Long‐term priming of hypothalamic microglia is associated with energy balance disturbances under diet‐induced obesity
Source: Glia. 2022 May 23;70(9):1734–61. doi: 10.1002/glia.24217 (PMC9540536; doi:10.1002/glia.24217)
Supplement: Supplementary file 1 — Appendix S1: Supporting Information. [file GLIA-70-1734-s001.pdf]

Table S1. Sequence of the primers used for qPCR

Gene	Forward (5´>3´)	Reverse (5´>3´)	Size	Accession #
GAPDH (rat)	TGGAGTCTACTGGCGTCTTC	CTAAGCAGTTGGTGGTGCAG	186	NM_017008.4
IBA1 (rat)	GGATCAACAAGCACTTCCTC	CTCCAGCATTCGCTTCAA	149	NM_017166.3
GFAP (rat)	TCGAGATCGCCACCTACAG	TTGTGCTCCTGCTTCGACT	205	NM_017009.2
IL-1β (rat)	GCCAACAAGTGGTATTCTCC	CCGTCTTTCATCACACAGGA	118	NM_031512.2
NLRP3 (rat)	GGGACTCAAGCTCCTCTGTG	GGCTCTGGTTATGGGTCAGA	131	NM_001191642.1
MHC II (rat)	GCTGGTCTGTTTCATCAGCA	GCGTCACAGTGCAGGAGTAA	185	NM_198741.2
Gal-3 (rat)	ACGACATCGCCTTCCACTTT	TGAATGGTTTGCCGCTCTCA	138	NM_031832.1
Trem2 (rat)	TCCTGTTGCTGGTCACAGAG	CTCCCATTCTGCTTCCTCAG	220	NM_001106884.1
Scarb1 (rat)	AGTAAAAAGGGCTCGCAGGA	CTTCTGGGCCCTACAGCTTG	112	NM_031541.1
Arg1 (rat)	TCGTACTGTGAACACGGCAG	GCCTGGTTCTGTTCGGTTTG	163	NM_017134.3
GAPDH (mouse)	TGAACGGGAAGCTCACTGG	TCCACCACCCTGTTGCTGTA	307	NM_008084.3
IBA1 (mouse)	GACTGCCAGCCTAAGACAAC	AAGGCTTCAAGTTTGGACGG	185	NM_001361501.1
GFAP (mouse)	CAACGTTAAGCTAGCCCTGGACAT	TCGGATCTGGAGGTTGGAGAA	112	NM_001131020.1
IL-1β (mouse)	GAGTGTGGATCCCAAGCAAT	ACGGATTCCATGGTGAAGTC	201	NM_008361.4
NLRP3 (mouse)	GCTAAGAAGGACCAGCCAGA	CAGCAAACCCATCCACTCTT	99	NM_145827.4
MHC II (mouse)	TCCTCAAGCGACTGTGTTCC	CGTCTGCGACTGACTTGCTA	134	NM_010378.3
Gal-3 (mouse)	TAATCAGGTGAGCGGCACAG	CGGATATCCTTGAGGGTTTGG	107	NM_001145953.1
Trem2 (mouse)	GAAAGTACTGGTGGAGGTGCT	CTTGATTCCTGGAGGTGCTGT	125	NM_031254.3
Scarb1 (mouse)	CTGAGCACGTTCTACACGCA	GGCCTGAATGGCCTCCTTAT	189	NM_016741.2
Arg1 (mouse)	TGAACACGGCAGTGGCTTTA	CATGTGGCGCATTCACAGTC	113	NM_007482.3
NLRP3 (mouse)	GCTAAGAAGGACCAGCCAGA	CAGCAAACCCATCCACTCTT	99	NM_145827.4
HMGB1 (mouse)	CCATTGGTGATGTTGCAAAG	CAGCTTGGCAGCTTTCTTCT	92	NM_001313894.1
POMC (mouse)	CATGACCTCCGAGAAGAGCC	GTGCGCGTTCTTGATGATGG	70	NM_001278584.1
NPY (mouse)	TGGACTGACCCTCGCTCTAT	TAGTGTCGCAGAGCGGAGTA	135	NM_023456.3
AgRP (mouse)	CTTTGGCGGAGGTGCTAGAT	TGCGACTACAGAGGTTCGTG	189	NM_001271806.1

Table S2. Description of the morphological parameters measured in microglial cells

Morphological parameter	Description
Fractal dimension (*D*)	Determined by the box counting method, is a recognized parameter to identify intermediate microglial forms ranging from simple rounded to complex branched. A higher D means a greater complexity of the pattern.
Lacunarity (Λ)	Measures heterogeneity or translational and rotational invariance in a shape. Lacunarity reflects the heterogeneity of the cell profile. High Λ measures mean heterogeneity of an image, which contains many differently sized gaps or lacunas.
Cell area	The total number of pixels present in the filled shape of the cell image, later transformed to squared micrometers.
Convex hull area (CHA)	The convex hull is the smallest convex polygon (that with all interior angles smaller than 180°) containing the whole cell shape. Thus, it is related to the cell area.
Density	Calculated by dividing the *area* of the cell by its *convex hull area*. A higher *density* indicates a more compact cell, or with less gaps.
Cell perimeter	The single outline cell shape expressed in microns. Also related to the cell size.
Convex hull perimeter (CHP)	The single outline of the convex hull expressed in microns.
Roughness	The ratio of *cell perimeter* to the *convex hull perimeter*. It reflects the extent of cell surface irregularities, being more independent of the cell size.
Convex hull of span ratio (CHSR)	The ratio of the major to the minor axes of the convex hull. Indicates an elongated shape.
Cell circularity (CC)	Calculated as (4π × *cell area*) / (*cell perimeter*)^2. The circularity value of a circle is 1. This parameter decreases as the cell profile moves away from a circular shape.
Convex hull circularity (CHC)	Calculated as (4π × *convex hull area*) / (*convex hull perimeter*)^2.
Bounding circle diameter (BCD)	Is the diameter (expressed in µm) of the smallest circle that encloses the convex hull. Also related to the cell size.
Maximum span across the convex hull (MSACH)	Is the maximum distance between two points across the convex hull. It is related to the size as well as to the elongated shape of a cell.
Ratio convex hull radii (RCHR)	Is the division of the largest to the smallest radius from the center of mass of the convex hull to an exterior point.
Mean radius	Calculated as the mean length in microns from the center of mass of the convex hull to an exterior point.

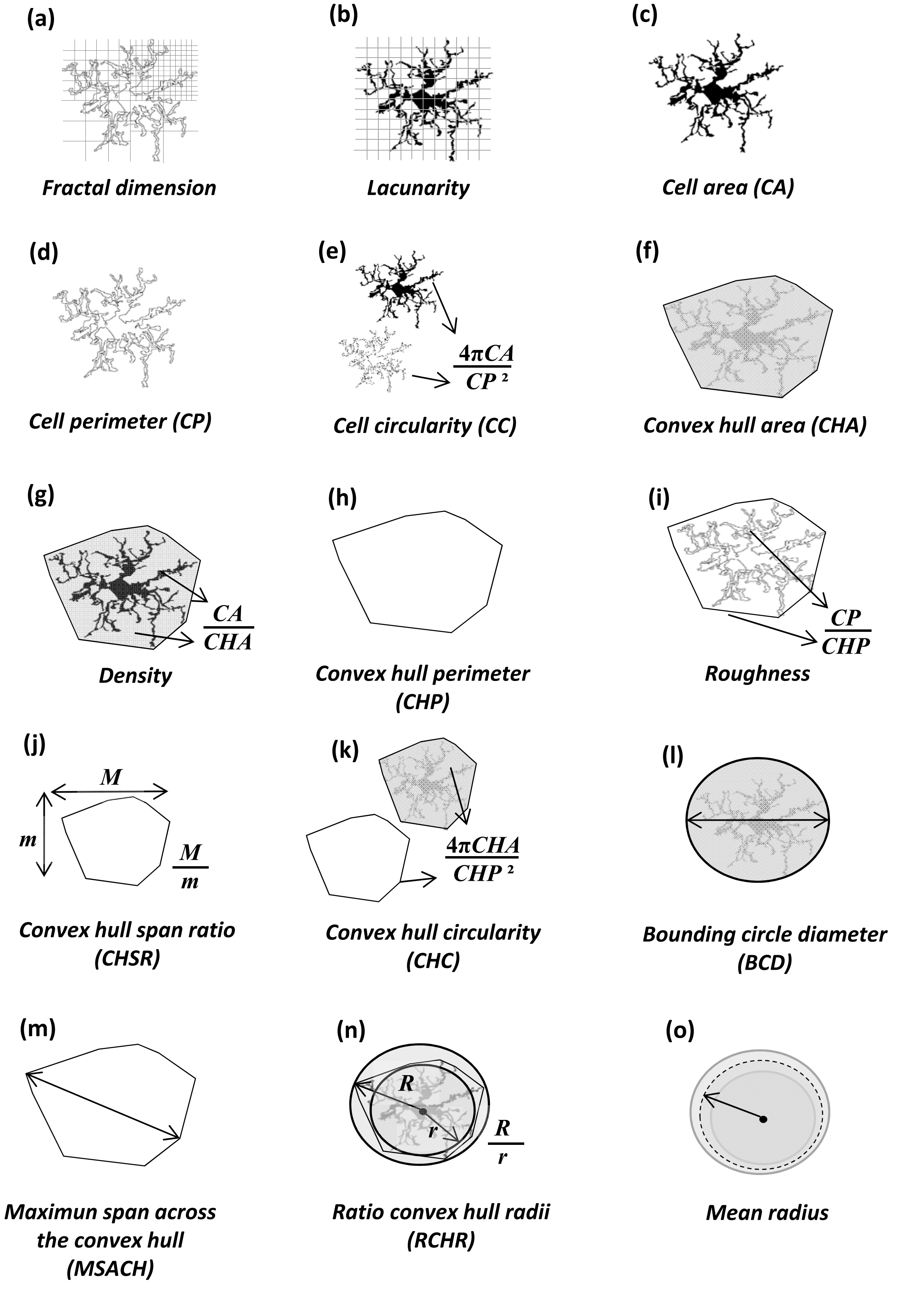

Figure S1. Schematic explanation of the 15 morphological parameters measured in microglial cells.

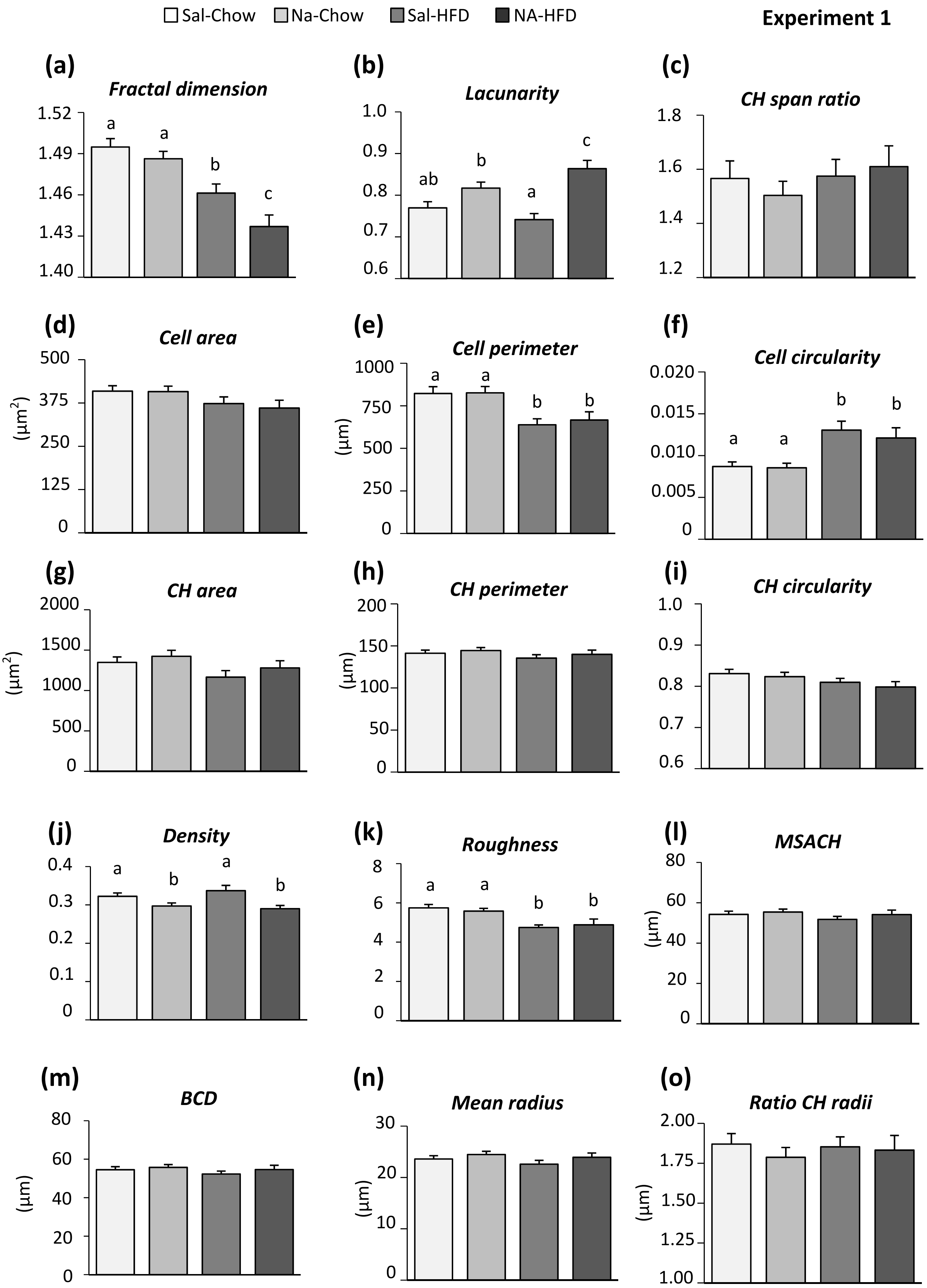
Sal-Chow
Na-Chow
Sal-HFD
NA-HFD
Experiment 1
(a)
Fractal dimension
1.52
1.49
1.46
1.43
1.40
a
a
b
c
(b)
Lacunarity
1.0
0.9
0.8
0.7
0.6
ab
b
a
c
(c)
CH span ratio
1.8
1.6
1.4
1.2
(d)
Cell area
(μm2)
500
375
250
125
0
(e)
Cell perimeter
(μm)
1000
750
500
250
0
a
a
b
b
(f)
Cell circularity
0.020
0.015
0.010
0.005
0
a
a
b
b
(g)
CH area
(μm2)
2000
1500
1000
500
0
(h)
CH perimeter
(μm)
200
150
100
50
0
(i)
CH circularity
1.0
0.9
0.8
0.7
0.6
(j)
Density
0.4
0.3
0.2
0.1
0
a
b
a
b
(k)
Roughness
8
6
4
2
0
a
a
b
b
(l)
MSACH
(μm)
80
60
40
20
0
(m)
BCD
(μm)
80
60
40
20
0
(n)
Mean radius
(μm)
30
20
10
0
(o)
Ratio CH radii
(μm)
2.00
1.75
1.50
1.25
1.00

Figure S2

Experiment 3

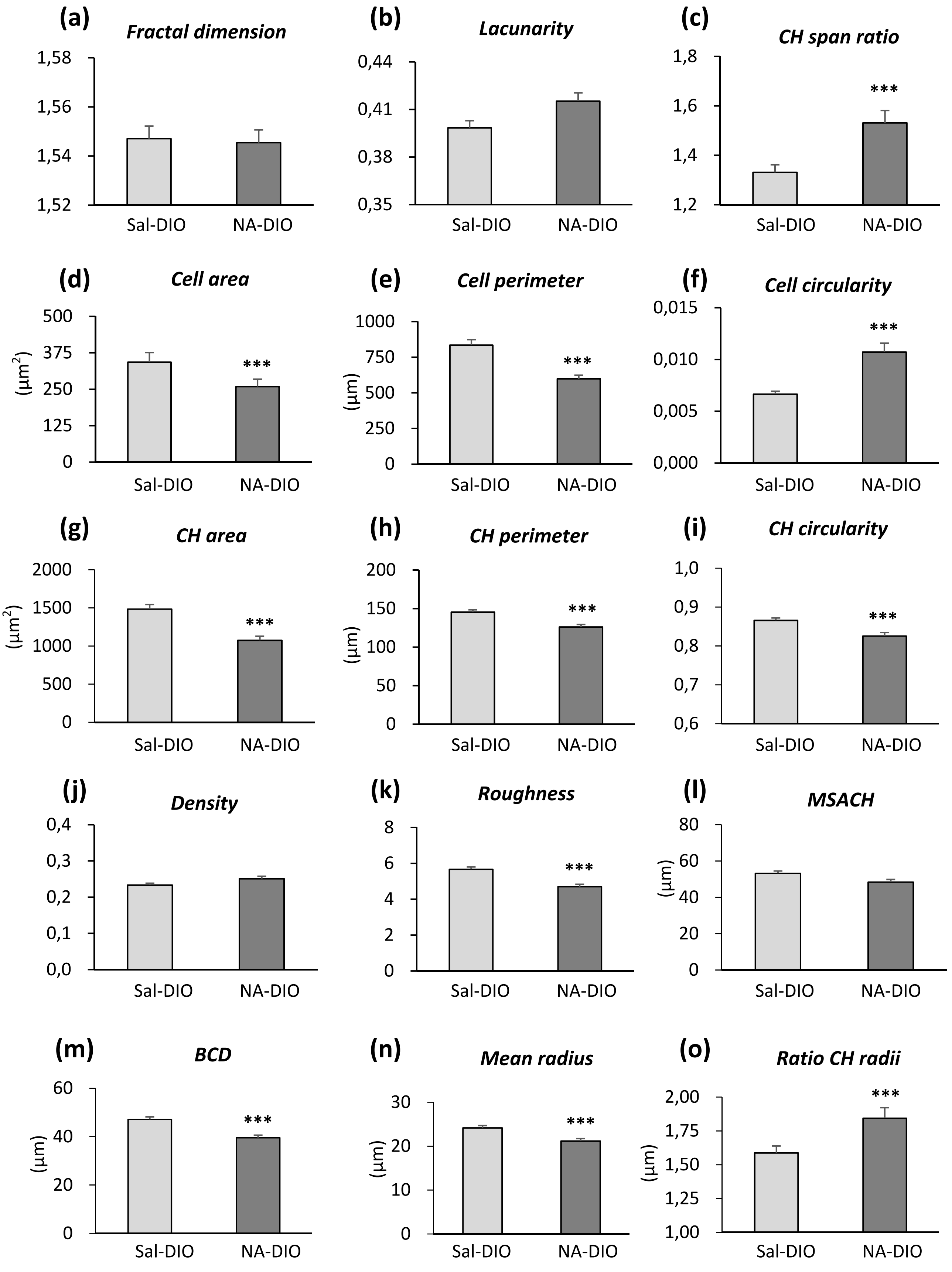

Figure S3

FIGURE S2. Morphological characterization of microglial cells in the arcuate nucleus of rats treated with two inflammatory stimuli applied three months apart. Rats were treated with two inflammatory stimuli (NA and HFD), applied three months apart, using saline (Sal) and standard chow respectively as controls for those stimuli (Experiment 1). Brain sections were immunostained for IBA1. Images of IBA1-positive microglia located in the arcuate nucleus were processed to obtain a binary image of the cell´s profile. These processed images were used to measure 15 morphometric parameters, which are listed and explained in Table S2 and Figure S1. The graphs present the mean ± SEM of these parameters measured in n=30-40 cells from each experimental group. Letters *a-c* above the bars denote if there is a statistical difference between groups: the same letter stands for no difference; different letters stand for a statistical difference ($P<0.05$). CH: convex hull.

FIGURE S3. Morphological characterization of microglial cells in the arcuate nucleus of mice ICV-injected with NA and later made obese by DIO. One month after receiving an ICV injection of NA, mice were fed a HFD for three months to induce obesity (Experiment 3). Brain sections were immunostained for IBA1. Images of IBA1-positive microglia located in the arcuate nucleus were processed to obtain a binary image of the cell´s profile. These processed images were used to measure 15 morphometric parameters (see Table S2 and Figure S1). The graphs present the mean ± SEM of these parameters measured in n=50 cells from each experimental group. ***$P<0.001$; CH: convex hull.
